# Melatonin improves influenza virus infection-induced acute exacerbation of COPD by suppressing macrophage M1 polarization and apoptosis

**DOI:** 10.1186/s12931-024-02815-0

**Published:** 2024-04-27

**Authors:** Meng-Meng Xu, Jia-Ying Kang, Qiu-Yan Wang, Xing Zuo, Yuan-Yuan Tan, Yuan-Yuan Wei, Da-Wei Zhang, Ling Zhang, Hui-Mei Wu, Guang-He Fei

**Affiliations:** 1 Department of Respiratory and Critical Care Medicine, The First Affiliated Hospital of Anhui Medical University, Hefei, Anhui 230022 China; 2 Key Laboratory of Respiratory Disease Research and Medical Transformation of Anhui Province, The First Affiliated Hospital of Anhui Medical University, Hefei, Anhui 230022 China; 3 Department of Pulmonary and Critical Care Medicine, The First Affiliated Hospital of USTC, Division of Life Sciences and Medicine, University of Science and Technology of China, Hefei, Anhui 230001 China; 4 Anhui Geriatric Institute, Department of Geriatric Respiratory Critical and Care Medicine, The First Affiliated Hospital of Anhui Medical University, Hefei, Anhui 230022 China; 5 Emergency Department, The First Affiliated Hospital of Anhui Medical University, Hefei, Anhui 230022 China; 6 Department of Critical Care Medicine, The First Affiliated Hospital of Anhui Medical University, Hefei, Anhui 230022 China

**Keywords:** Melatonin, Chronic obstructive pulmonary disease, Influenza virus, Macrophage polarization, Apoptosis, Interleukin-1β

## Abstract

**Background:**

Influenza A viruses (IAV) are extremely common respiratory viruses for the acute exacerbation of chronic obstructive pulmonary disease (AECOPD), in which IAV infection may further evoke abnormal macrophage polarization, amplify cytokine storms. Melatonin exerts potential effects of anti-inflammation and anti-IAV infection, while its effects on IAV infection-induced AECOPD are poorly understood.

**Methods:**

COPD mice models were established through cigarette smoke exposure for consecutive 24 weeks, evaluated by the detection of lung function. AECOPD mice models were established through the intratracheal atomization of influenza A/H3N2 stocks in COPD mice, and were injected intraperitoneally with melatonin (Mel). Then, The polarization of alveolar macrophages (AMs) was assayed by flow cytometry of bronchoalveolar lavage (BAL) cells. In vitro, the effects of melatonin on macrophage polarization were analyzed in IAV-infected Cigarette smoking extract (CSE)-stimulated Raw264.7 macrophages. Moreover, the roles of the melatonin receptors (MTs) in regulating macrophage polarization and apoptosis were determined using MTs antagonist luzindole.

**Results:**

The present results demonstrated that IAV/H3N2 infection deteriorated lung function (reduced FEV_20,50_/FVC), exacerbated lung damages in COPD mice with higher dual polarization of AMs. Melatonin therapy improved airflow limitation and lung damages of AECOPD mice by decreasing IAV nucleoprotein (IAV-NP) protein levels and the M1 polarization of pulmonary macrophages. Furthermore, in CSE-stimulated Raw264.7 cells, IAV infection further promoted the dual polarization of macrophages accompanied with decreased MT1 expression. Melatonin decreased STAT1 phosphorylation, the levels of M1 markers and IAV-NP via MTs reflected by the addition of luzindole. Recombinant IL-1β attenuated the inhibitory effects of melatonin on IAV infection and STAT1-driven M1 polarization, while its converting enzyme inhibitor VX765 potentiated the inhibitory effects of melatonin on them. Moreover, melatonin inhibited IAV infection-induced apoptosis by suppressing IL-1β/STAT1 signaling via MTs.

**Conclusions:**

These findings suggested that melatonin inhibited IAV infection, improved lung function and lung damages of AECOPD via suppressing IL-1β/STAT1-driven macrophage M1 polarization and apoptosis in a MTs-dependent manner. Melatonin may be considered as a potential therapeutic agent for influenza virus infection-induced AECOPD.

**Graphical Abstract:**

Schematic mechanisms underlying the regulatory effects of melatonin on macrophage polarization and apoptosis in IAV infection plus cigarette stimulation-induced AECOPD model.
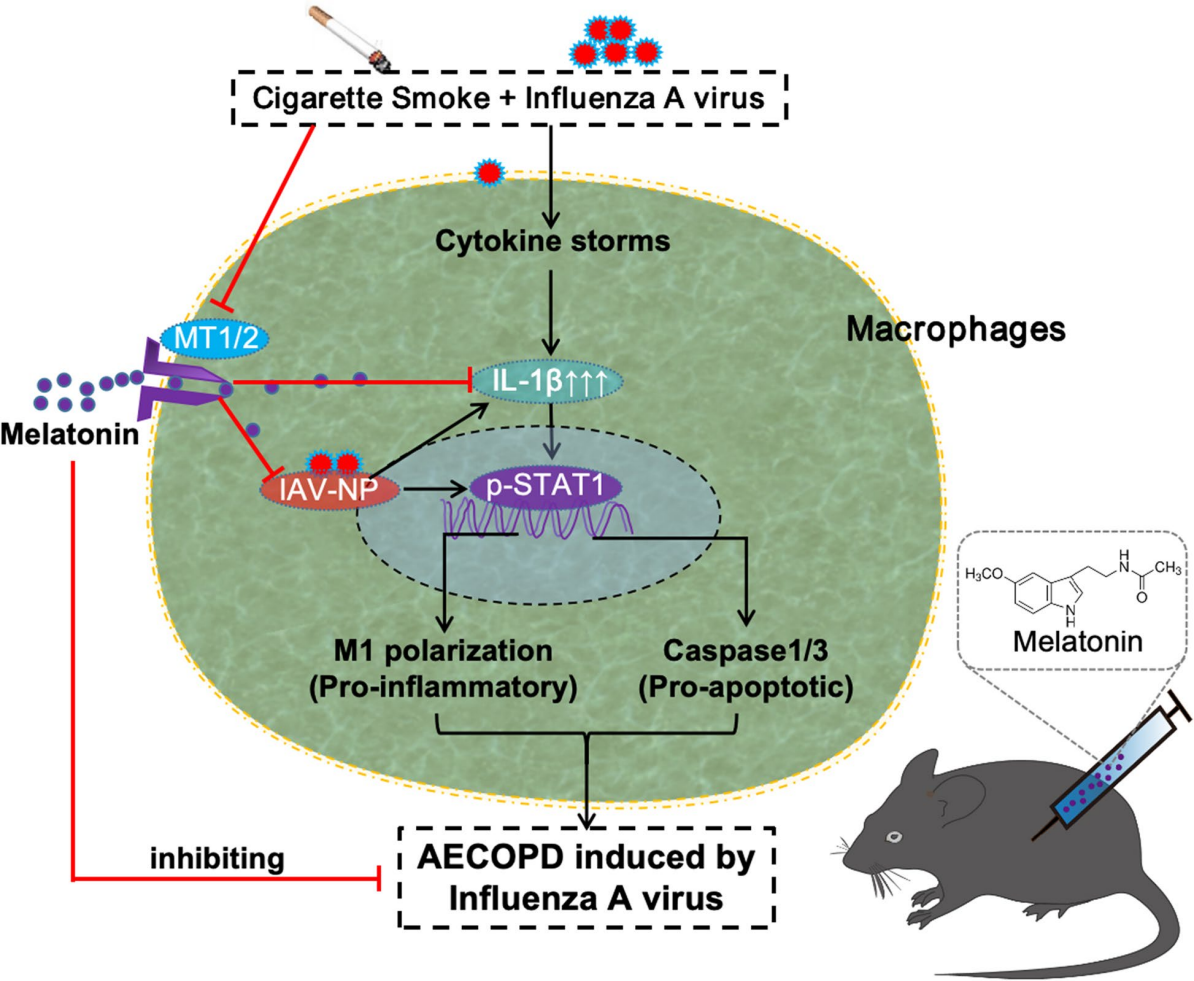

**Supplementary Information:**

The online version contains supplementary material available at 10.1186/s12931-024-02815-0.

## Introduction

Influenza virus infection, a extremely harmful public health problem, causes huge disease burden worldwide. World Health Organization (WHO) estimated that seasonal influenza affected 57% global populations, caused approximately 3–5 million severe cases and 290,000–650,000 respiratory deaths annually [1, 2], especially among the older adults with chronic obstructive pulmonary disease (COPD) [3, 4]. Our previous study confirmed that influenza A virus (IAV) was the most common respiratory virus associated with acute exacerbation of COPD (AECOPD) in Asia [5].

Influenza viruses are primarily divided into three types (A, B and C), in which influenza viruses A and B are prone to cause seasonal influenza epidemics [6]. Currently, type A influenza viruses (H1N1 and H3N2) are accounted for most influenza epidemics worldwide. In mainland China, influenza A/H3N2 subtypes exhibited the longest mean epidemic duration of approximately 4.83 months/year, followed by influenza virus B and A/H1N1 [2, 7, 8]. Besides, influenza A/H3N2-associated excess respiratory deaths were the highest, 80% of which occurred in people aged ≥ 60 years [9]. Therefore, it is of great importance to explore the potential molecular mechanisms and effective therapeutic agents directing against IAV infection-triggered AECOPD.

Influenza virus infection is extremely prone to induce lung epithelium injuries and leukocyte infiltration, resulting in airway cytokine storms or “hypercytokinemia” [10]. As highly plastic innate immune cells, macrophages form the first immune lines against viruses and bacteria infection. Macrophages can be polarized into multi-functional phenotypes depending on different stimulus [11]. Cigarette irritation causes the polarization of pulmonary macrophages into classically activated type (M1 type), which is commonly triggered by signal transducer and activator of transcription 1 (STAT1) signaling [12, 13]. Activated STAT1 signaling drives the expression of signature M1 genes such as inducible nitric oxide synthase (iNOS), CD86, monocyte chemoattractant protein 1 (MCP1) and interleukin-1β (IL-1β), which cause pulmonary inflammatory injuries, even apoptosis, exacerbate pulmonary damages of COPD [14]. And, alternatively activated macrophages (M2 type) are also closely related with pulmonary damages of COPD [14, 15]. M2 alveolar macrophages (AMs) are commonly evoked through signal transducer and activator of transcription 6 (STAT6) signaling, which drives the expression of M2 markers (IL-4, CD206, Arginase 1 (Arg1) and TGF-β, etc.) with the formation of fibroproliferative microenvironments. The difference of airway microenvironments determines the proportion of M1 and M2 macrophages, also affects the process of “injuries-repairments” of lung tissues [16, 17]. Our recent study revealed that acute IAV infection induced acute lung injury (ALI) via enhanced the M1 polarization of pulmonary macrophages, and inhibiting M2 polarization [18]. However, in cigarette smoke-exposed lung tissues, the effects of in acute IAV infection on pulmonary macrophage polarization remain to be clarified.

The neurohormone melatonin (N-acetyl-5-methoxytryptamine) is primarily synthesized and released by pineal gland at night, acts on two high-affinity G protein-coupled receptors, the melatonin receptors (MT1 and MT2) [19, 20]. Melatonin accounts for the modulation of sleep and circadian rhythm in brains. In the periphery, melatonin exerts anti-inflammatory and anti-oxidative effects through its receptors [21, 22]. In lung tissues, melatonin may exert beneficial effects through regulating macrophage polarization as well as the expression of pro-inflammatory and pro-apoptotic proteins, such as STAT1/6 signaling as well as NLRP3 and Caspase 1/3 proteins [23]. Clinical studies showed a decreased trend in serum melatonin levels of COPD patients, and a further decrease occured with the exacerbation of COPD symptoms [24, 25]. Specifically, serum melatonin levels were positively correlated with lung function (FEV_1_/FVC, FEV_1_% pred), as well as antioxidant enzymes (SOD, CAT and GSH-Px) [24, 25]. In COPD rats, melatonin inhibited pulmonary apoptosis and endoplasmic reticulum (ER) stress by decreasing Caspase 3/12 and increasing SIRT1 expression [26]. Moreover, melatonin is also capable to resist virus infection, such as influenza virus and severe acute respiratory syndrome coronavirus 2 (SARS-CoV-2) infection [27, 28]. Our recent study demonstrated that melatonin alleviated IAV infection-induced ALI by inhibiting the M1 polarization of AMs with the decreased expression of iNOS, MCP1 and CD86 [18]. However, the effects of melatonin on IAV infection-induced AECOPD remain unknown.

Therefore, this study aims to investigate whether melatonin exerts the effects of anti-influenza virus infection, further improves IAV infection-induced pulmonary acute damages in cigarette smoke-induced COPD mice, and to identify the underlying signal transduction mechanisms of melatonin which underpin its protective effects against influenza virus infection-induced COPD.

## Materials and methods

### Experimental animals

Male C57BL/6 mice (8 weeks old) were housed in the Laboratory Animal Research Center of Anhui Medical University under standard specific pathogen-free condition with 12-h light/12-h dark cycles at 22 ± 2 °C, and free access to the standard laboratory rodent diets and water during modeling experiments. All experimental mice were treated according to the protocols approved by the Animal Care and Ethics Committee of Anhui Medical University (Approval no.20210056, no.LLSC20221233).

### Influenza A/H3N2 amplification and plaque assay

Influenza virus A/Anhui/1/2017 (H3N2) was obtained from Prof. Yan Liu (Department of Microbiology, Anhui Medical University, China), and isolated from the patients infected influenza A/H3N2 in 2017, and used in laboratory studies strictly according to standard bio-safety operation practices. The IAV samples were amplified in Madin-Darby canine kidney (MDCK) cells and specific pathogen-free embryonated chicken eggs. Virus titers were assayed via the standard plaque assay on MDCK cells according to previous description [18, 29]. MDCK cells were infected with diluted virus samples 1–2 h at 37 °C. After washed with PBS, the cultivation was proceeded in the plaque medium containing 50% 2 × DMEM, 50% avecil (2.35%) and N-acetyl trypsin (1.5 μg/ml) for 72 h. Then, the cells were stained with Neutral Red stain, and plaques were counted for the calculating virus titers. All experiments involved in viruses were performed according to the bio-safety level two requirements with well-equipped personal protection for all the researchers.

### Animal models of AECOPD induced by influenza A/H3N2 virus

Mice were randomly divided into 4 groups (10 mice each group): 1) air group; 2) cigarette smoke (CS) exposure-induced COPD group; 3) IAV/H3N2 infection-induced acute exacerbation of COPD (AECOPD) group; 4) AECOPD + melatonin group. To establish COPD model, mice were exposed whole bodies to CS in a passive smoking chamber (70 cm × 40 cm × 60 cm) with a house directing flow inhalation and CS-exposure system containing in a laminar flow and CS extraction units. Regular CS exposure was proceeded with 10 cigarettes per run, twice/day, 6 days/week for up to 24 weeks. Control mice were exposed to normal air. After 24 h of final CS exposure, mice were anesthetized with 1% sodium pentobarbital (50 mg/kg, ip) free from pain for invasive trachea cannula. To establish AECOPD models, mice were atomized intratracheally with 50 μl IAV/H3N2 stock (100 plaque forming units, PFUs) on day 0 and day 3, while the air mice were atomized intratracheally with 50 μl saline. From day 0, mice were injected intraperitoneally with saline or melatonin (Mel) in 5% DMSO (30 mg/kg) (C_13_H_16_N_2_O_2_, stated purity ≥ 98%, M5250, Sigma-Aldrich, USA) at daily 18:00 for consecutive 7 days.

### Lung function detection

After anesthesia with an intraperitoneal injection of 1% pentobarbital (Injection dose (ml) = mouse body weight (g) × 4/1000), mice were tracheostomized and placed in a whole-body plethysmograph of PFT Pulmonary Maneuvers (DSI Buxco, Minnesota, USA). Total lung capacity (TLC), functional residual capacity (FRC), static lung compliance (chord compliance, Cchord) were measured from the quasi-static pressure-volume (PV) maneuver. Forced vital capacity (FVC), volume expired in first 20 and 50 ms (ms) of fast expiration (FEV_20_, FEV_50_) were measured from the fast-flow volume (FV) maneuver. Inspiratory time (Ti), expiratory time (Te), peak inspiratory flow (PIF) and minute volume (MV) were recorded during resistance and compliance (RC) maneuver.

### Histological analysis and Lm evaluation

Mice were euthanatized by high-dose 1% sodium pentobarbital (100 mg/kg, ip), the left lung lobes were dissected without proceeding bronchoalveolar lavage, and fixed with 4% paraformaldehyde, and embedded in paraffins. Four micrometers sections were stained with haematoxylin and eosin (H&E) for evaluating the severities of lung injury and emphysema. The indexes of lung injury were double-blindly calculated according to the scoring system including five histological features: a. neutrophils in the alveolar space; b. neutrophils in the interstitial space; c. hyaline membranes formation; d. proteinaceous debris filling the airspace; e. alveolar septal thickening. Each item was scored 0, 1, or 2 based on the severity of lung injury. The final injury scores were figured up according to the following formula [30],

$$\mathrm{Lung\, injury \,Scores}\hspace{0.17em}=\hspace{0.17em}[(20\hspace{0.17em}\times \hspace{0.17em}{\text{a}})\hspace{0.17em}+\hspace{0.17em}(14\hspace{0.17em}\times \hspace{0.17em}{\text{b}})\hspace{0.17em}+\hspace{0.17em}(7\hspace{0.17em}\times \hspace{0.17em}{\text{c}})\hspace{0.17em}+\hspace{0.17em}(7\hspace{0.17em}\times \hspace{0.17em}{\text{d}})\hspace{0.17em}+\hspace{0.17em}(2\hspace{0.17em}\times \hspace{0.17em}{\text{e}})] / (\mathrm{number\, of\, fields}\hspace{0.17em}\times \hspace{0.17em}100).$$

Pulmonary emphysema is evaluated by measuring the mean linear intercept (Lm) which shows interalveolar septal wall distances. The ocular micrometer with 5 lines (each 500 μm long) is utilized to determine Lm of alveolars (avoiding the fields with airways or vessels).

### BALF collection and leukocytes counting

After complete anesthesia with 0.2 ml 1% sodium pentobarbital (100 mg/kg, ip), bronchoalveolar lavage was performed with 2 ml sterile PBS via an endotracheal tube. The bronchoalveolar lavage fluids (BALF) were centrifuged at 4 ºC, 1000 rpm for 10 min. Cell pellets were re-suspended in PBS with Red Blood Cell (RBC) Lysis Buffer (C3702, Beyotime technology, Shanghai, China), for total leukocytes counting using a hemocytometer. Then, smeared BAL cells were stained with Wright-Giemsa stain solution (Baso Diagnostics Inc, Zhuhai, China).

### Flow cytometry analysis of BALF cells

BAL cells were treated with RBC lysis buffer, and stained with the following fluorochrome-conjugated antibodies to screen alveolar macrophages (AMs): CD45 (APC-Cy^TM^7, 561037, BD Biosciences, USA), CD11c (PerCP-Cy^TM^5.5, 561114, BD), Siglec-F (BV421, 565934, BD). For investigating the effects of melatonin on AMs polarization, BALF cells were stained with M1 macrophage marker CD86 (PE, 561963, BD) and M2 marker CD206 (MR6F3 APC, ThermoFisher Scientific, USA). Specifically, before transmembrane protein CD206 stained, BALF cells were fixed and permeabilized for better intracellular staining. The images and data of flow cytometry were collected using LSRFortessa X30 (BD Biosciences, USA).

### Cigarette smoking extract (CSE) preparation

In brief, one burning cigarette (Marlboro Red Label, Longyan Tobacco Industrial co. LTD, Fujian, China) without the filter is sucked at a continuous steady flow-rate (8 ml/s, 40 ml cigarette smoking) into a 50 ml syringe containing 10 ml PBS, and mixing upside down with PBS for 1 min, repeated for 5 times so that the cigarette just finished burning. Next, adjust its pH to 7.4 and filter it with a 0.22 µm filter (Millipore, Bedford, MA). The final concentration is considered as 100%. The obtained CSE was packaged and stored in the -80℃ refrigerator for using in cell experiments.

### Cell culture and treatment

Raw264.7 macrophage lines were purchased from Cell Bank of Shanghai Institutes for Biological Sciences (China Academy of Science, Shanghai, China). Raw264.7 cells were cultured in Dulbecco’s modified Eagle’s medium (DMEM) (Hyclone, Logan, UT, USA) with 10% foetal bovine serum (FBS) (Excell biology) at 37 °C under saturated humidity conditions containing 5% CO2. When growing to 60–70% confluence, according to previous studies and cell viability analysis [31–33], 3% CSE and IAV (Multiplicity of Infection, MOI = 2), melatonin (10 μM, 100 μM and 200 μM), luzindole (10 μM) and VX765 (50 μM) and IL-1β (10 ng/ml) were used in this experiment.

### Immunofluorescence staining in vivo and in vitro

The polarization of mouse pulmonary macrophages were detected through Immunofluorescence homologous double-labeling staining of 4 μm paraffin sections. The sections were stained with corresponding anti-rabbit primary antibodies: iNOS (1:1000, ab178945, abcam, USA) and CD206 (1:1000, ab300621, abcam) after antigen retrieval and BSA blocking. After reacted with HRP conjugated Goat Anti-Rabbit IgG, the sections were eluted with tyramide signal amplification (TSA) dye, and processed with microwaves. Next, the sections were stained with the anti-rabbit second antibodies: F4/80 (1:500, GB113373, Servicebio, Wuhan, China), and then stained with Cy3 conjugated goat anti-rabbit IgG, followed by nuclei staining with DAPI. Fluorescent images of the sections were captured by the laser-scanning confocal microscope (Zeiss LSM880, Carl Zeiss AG, Germany).

Raw264.7 cells were fixed with 4% paraformaldehydes, followed by permeabilization and blocking with 5% bull serum albumin (BSA). Then, the cells were incubated with primary antibodies against anti-rabbit Arg1 (1:50, #93668S, Cell signaling technology), anti-rabbit iNOS (1:200, ab178945, abcam), anti-mouse MT-1/2 ((1:50, sc-398788, Santa Cruz, USA) and anti-mouse influenza A nucleoprotein (IAV-NP) (1:100, sc-101352, Santa Cruz) overnight at 4 ℃. After the cells reacting with corresponding second antibodies (1:500, Alexa Fluor® 488 goat anti-rabbit IgG and Alexa Fluor® 594 goat anti-mouse IgG, Abcam), anti-fade Mounting Medium with DAPI (P0131, Beyotime technology) was applied to visualize nuclei. Fluorescent images were gained by the laser-scanning confocal microscope (Zeiss LSM880).

### Apoptosis detection

The apoptosis levels of Raw264.7 cells were detected using Annexin V-FITC/PI apoptosis detection kit (40302, Yeasen, Shanghai, China). After digested with trypsin without EDTA, the treated cells were collected and resuscitated with 100 μl 1 × Binding Buffers. And, 5 μl Annexin V-FITC and 10 μl PI Solution were added to stain apoptotic cells. After incubating 10 min avoiding lights, 400 μl 1 × Binding Buffers were added and the ratio of apoptotic cells (Annexin V+/PI+) was detected using LSRFortessa X30 (BD Biosciences).

### Reverse transcription-polymerase chain reaction (RT-PCR)

Total RNA was isolated with Trizol reagents (Invitrogen, USA). Reverse transcription was conducted using a 5 × Hieff™ One Step RT SuperMix with gDNA Remover (Yeasen, Shanghai, China) according to the manufacturer instruction. RT-PCR was performed using a Hieff™ Universal qPCR SYBR Master Mix (Yeasen). All samples were assayed in triplicates, and the target gene expression was normalized to β-actin. Relative mRNA expression was calculated with the 2^−∆∆Ct^ method. The specific primers for β-actin, melatonin receptor (MT) 1, MT2, IL-1β, Tumor Necrosis Factor α (TNF-α), monocyte chemoattractant protein 1 (MCP1), Arg1, Found in inflammatory zone 1 (Fizz1), IL-6, IL-18 and interferon γ (IFN-γ) were generated by Tsingke Biotech, Beijing, China. The primer sequences were listed in Supplementary Table 1.

### Western blot analysis

Total proteins were extracted with RIPA lysis containing protease inhibitors from mouse lung tissues and Raw264.7 cells. And, protein samples were separated through 10–13% SDS-PAGE, transferred to PVDF membranes. The membranes were incubated with primary antibodies: total-STAT1 (1:1000, PTM-5754, PTM Bio, Hangzhou, China), Phospho-STAT1 (1:1000, 340797, ZenBio, Chengdu, China), total-STAT6 (1:1000, 380957, ZenBio), Phospho-STAT6 (1:500, sc-136019, Santa Cruz), iNOS (1:1000, ab178945, Abcam), Arg1 (1:1000, #93668S, Cell signaling technology), Caspase1 (1:500, sc-392736, Santa Cruz), MT1/2 (1:500, sc-398788, Santa Cruz), β-Tublin (1:5000, M20005, Abmart, Shanghai, China) and GAPDH (1:5000, ab181602, Abcam) overnight at 4℃. After incubated with Goat Anti-Rabbit IgG second antibody (1:5000, M21003, Abmart), the membranes were visualized by Odyssey infrared imaging system (Tanon, Shanghai, China).

### Statistical analysis

All experiments were randomized and blinded. All results were presented as mean ± SD from at least three independent samples or biological replicates (n ≥ 3). Statistical analysis was performed using GraphPad Prism 9.0 (GraphPad Software, Inc., San Diego, CA). *Student’s t* tests were performed for comparisons between two different groups. One-way ANOVA with Bonferroni’s post hoc tests (for equal variance) or Dunnett’s T3 post hoc tests (for unequal variance) were performed for comparisons among multiple groups. ^*^p < *0.05* was considered statistically significant.

## Results

### Melatonin improved lung function of IAV-infected AECOPD mice

**Fig. 1:**
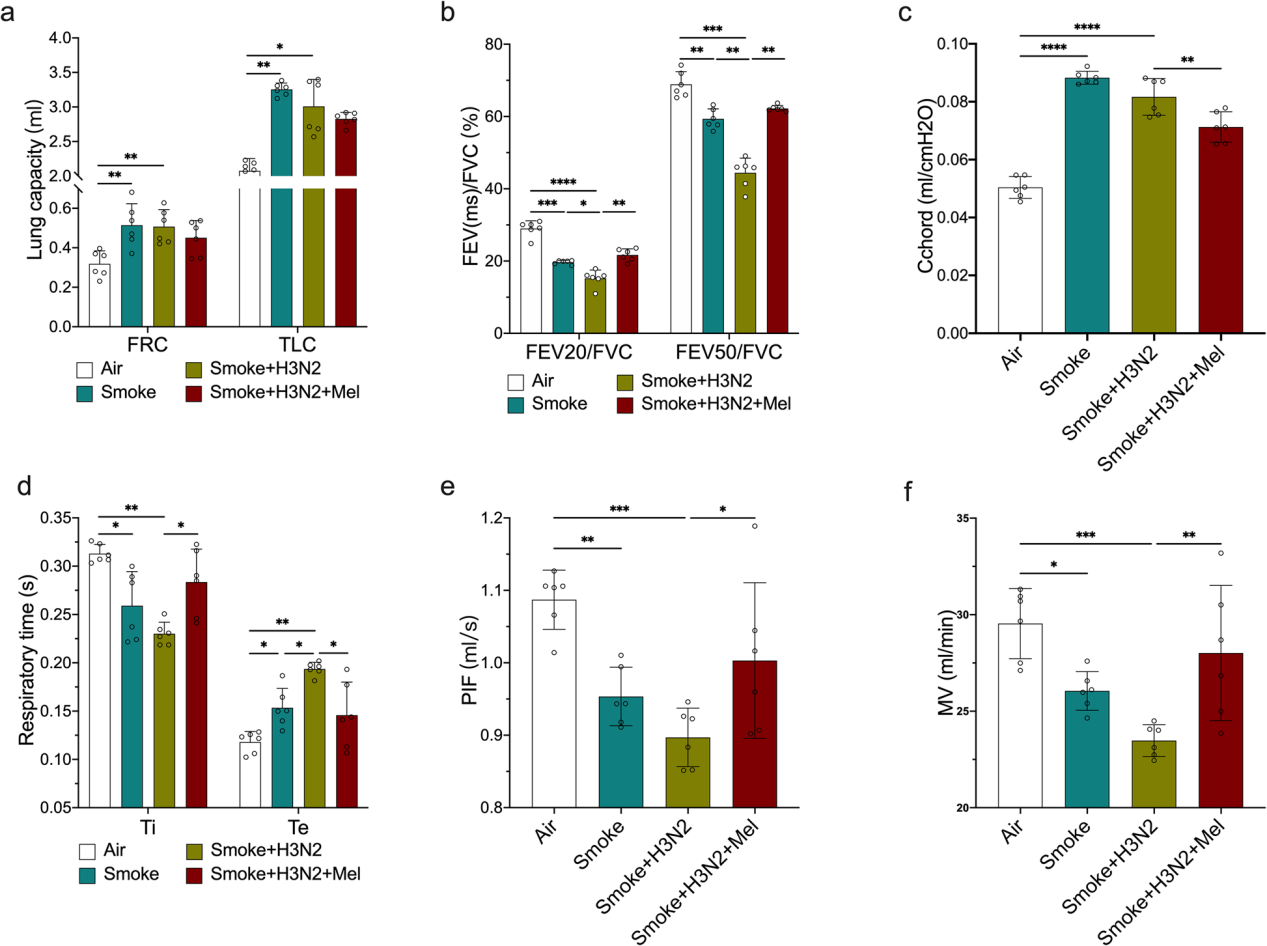
The regulatory impacts of melatonin on lung function of AECOPD mice induced by IAV infection. The chronic obstructive pulmonary disease (COPD) mouse model was established via cigarette smoke (CS) exposure for consecutive 24 weeks, and AECOPD mouse model was established through 1-week influenza virus A/H3N2 infection, verified through lung function detection. Individual values of total lung capacity (TLC) and functional residual capacity (FRC) (**a**), volume expired in first 20 and 50 ms of fast expiration (FEV_20_, FEV_50_) (**b**), static lung compliance (Cchord) (**c**), inspiratory time (Ti) and expiratory time (Te) (**d**), peak inspiratory flow (PIF) (**e**), and minute volume (MV) (**f**) from air group, COPD (smoke) group, AECOPD (smoke + H3N2) group, AECOPD + melatonin (Mel, 30 mg/kg) group. Data expressed as mean ± SD (*n* = 6). ^*^p < 0.05, ^**^p < 0.01, ^***^p < 0.001, ^****^p < *0.0001*

To verify whether COPD model was successfully established, mouse lung function was detected. There were significant increases in lung capacity parameters (FRC and TLC) and static compliance (Cchord), and decreases in respiratory airflow during expiration (FEV_20_/FVC, FEV_50_/FVC) in CS-exposed mice compared to air-exposed group (Fig. 1a-c), in line with lung function changes of COPD. CS exposure also increased expiratory times (Te), decreased inspiratory times (Ti), PIF and MV. IAV/H3N2 infection further decreased FEV_20_/FVC, FEV_50_/FVC, Ti, PIF and MV (Fig. 1b and d-f), indicating the exacerbation of respiratory airflow limitation in COPD mice. However, melatonin treatments decreased Cchord, calibrated respiratory times (Ti and Te), improved airflow limitation reflected by elevated FEV_20_/FVC, FEV_50_/FVC, PIF and MV (Fig. 1b-f). The results suggested that melatonin treatments had beneficial effects on lung function of AECOPD mice induced by IAV infection.

### Melatonin attenuated AECOPD induced by IAV infection

**Fig. 2:**
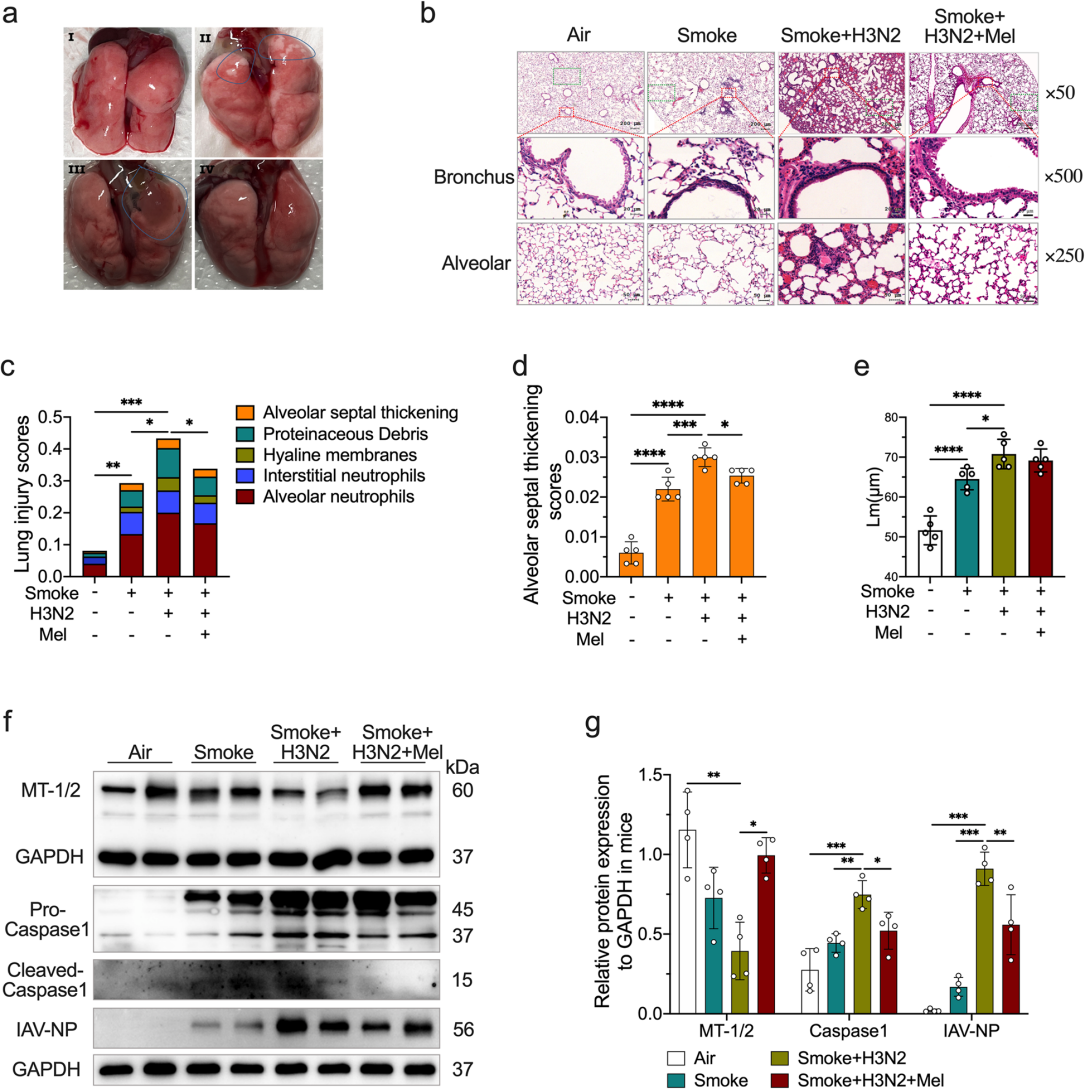
The protective effects of melatonin on lung damages of AECOPD mice. **a** Representative lung morphology of mice from air group, COPD (CS exposure) group, AECOPD (smoke + H3N2) group, AECOPD + melatonin (Mel, 30 mg/kg) group. Representative pulmonary alveoli and bronchial photomicrographs of mouse lung tissues in H&E-stained sections (**b**), individual values of lung injury scores (**c**), alveolar septal thickening scores (**d**) and the mean linear intercept (Lm) (**e**) of alveolar from each group (original magnification × 50, × 250 and × 500). Western blot analysis of the expression of MT1/2 (**f**), IAV-NP, Caspase1 (**g**) to GAPDH in lung tissue homogenates from each group. Data expressed as mean ± SD (*n* ≥ 3). ^*^p < 0.05, ^**^p < 0.01, ^***^p < 0.001, ^****^p < *0.0001*

Lung morphology showed that CS exposure for consecutive 24 weeks induced local pulmonary emphysema (Fig. 2a), an evident COPD phenotype. As shown in HE staining, CS exposure resulted in significant pulmonary alveoli enlargements with elevated alveolar mean linear intercepts (Lm), which were coupled with pulmonary leukocytes infiltration and airway wall thickening (Fig. 2b-e). H3N2 infection further exacerbated lung damages, enlarged Lm with local pulmonary edema as indicated in lung morphology in CS-exposed mice (Fig. 2a-e). Compared to air-exposed mice, CS exposure plus H3N2 infection decreased MT-1/2 expression (Fig. 2f-g). Intraperitoneal administration of melatonin in H3N2-infected COPD mice alleviated lung damages with decreased pulmonary leukocytes infiltration and alveolar septal thickness (Fig. 2b-d). In CS-exposed mice, H3N2 infection further up-regulated Caspase1 expression, which was inhibited by melatonin treatments (Fig. 2f-g). Melatonin also decreased IAV-NP expression, indicating its inhibitory effect on H3N2 infection (Fig. 2f-g).

### IAV infection promoted dual polarization of pulmonary macrophages in CS-induced COPD mice

**Fig. 3:**
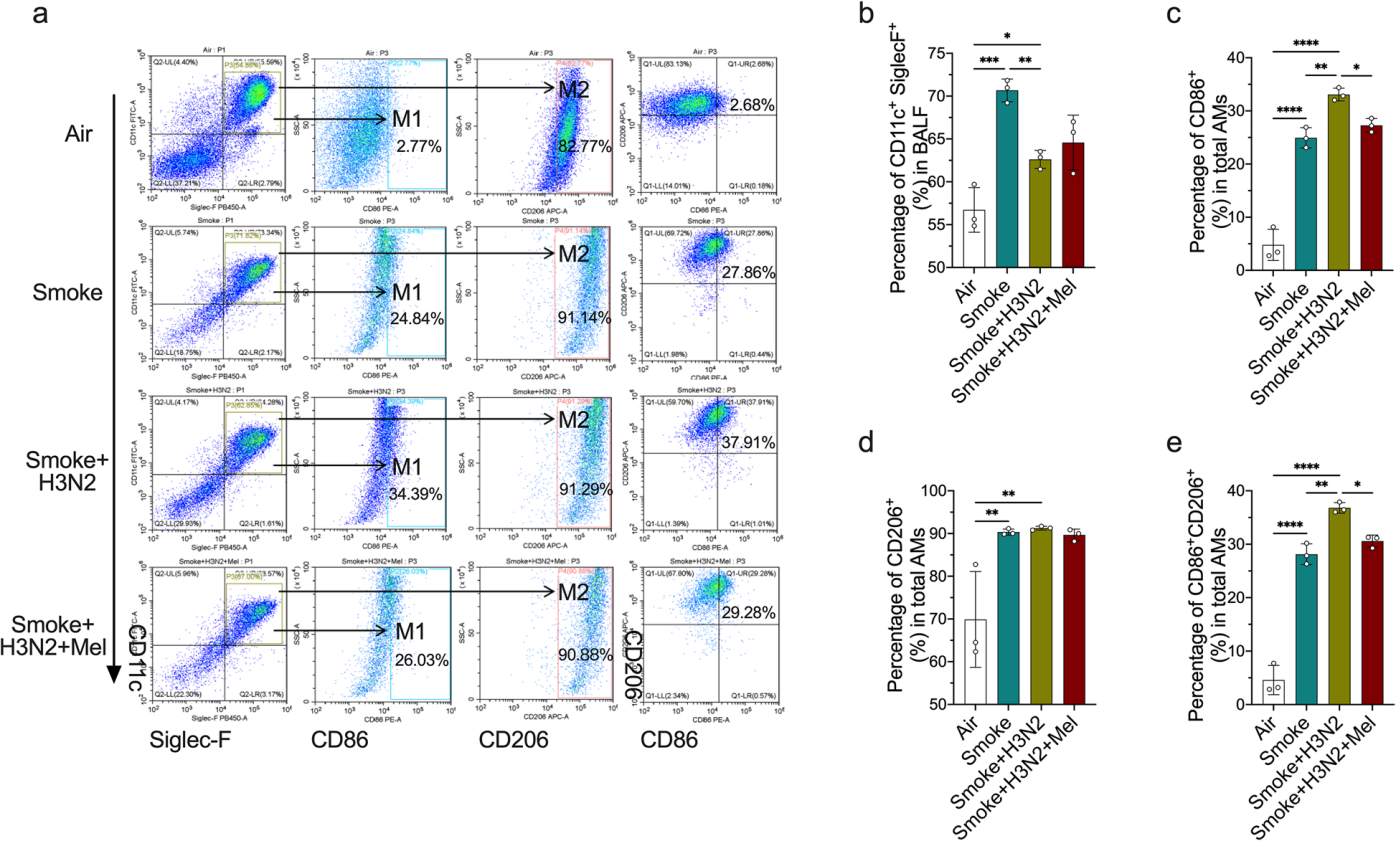
The effects of melatonin on abnormal polarization of AMs induced by cigarette exposure combined with IAV/H3N2 infection. **a** Voltage-gated strategy of flow cytometry analysis to identify alveolar macrophages (AMs) (CD45^+^Siglec-F^+^CD11c^+^) as well as CD86^+^ AMs (M1 type) and CD206^+^ AMs (M2 type) in BALF from from air group, COPD (smoke) group, AECOPD (smoke + H3N2) group, AECOPD + melatonin (Mel, 30 mg/kg) group. Individual percentages of Siglec-F + CD11c + AMs (**b**), CD86 + AMs (**c**), CD206 + AMs (**d**) and CD86 + CD206 + AMs (**e**) in BALF from each group. Data expressed as mean ± SD (*n* = 3). ^*^p < 0.05, ^**^p < 0.01, ^***^p < 0.001, ^****^p < *0.001*

**Fig. 4:**
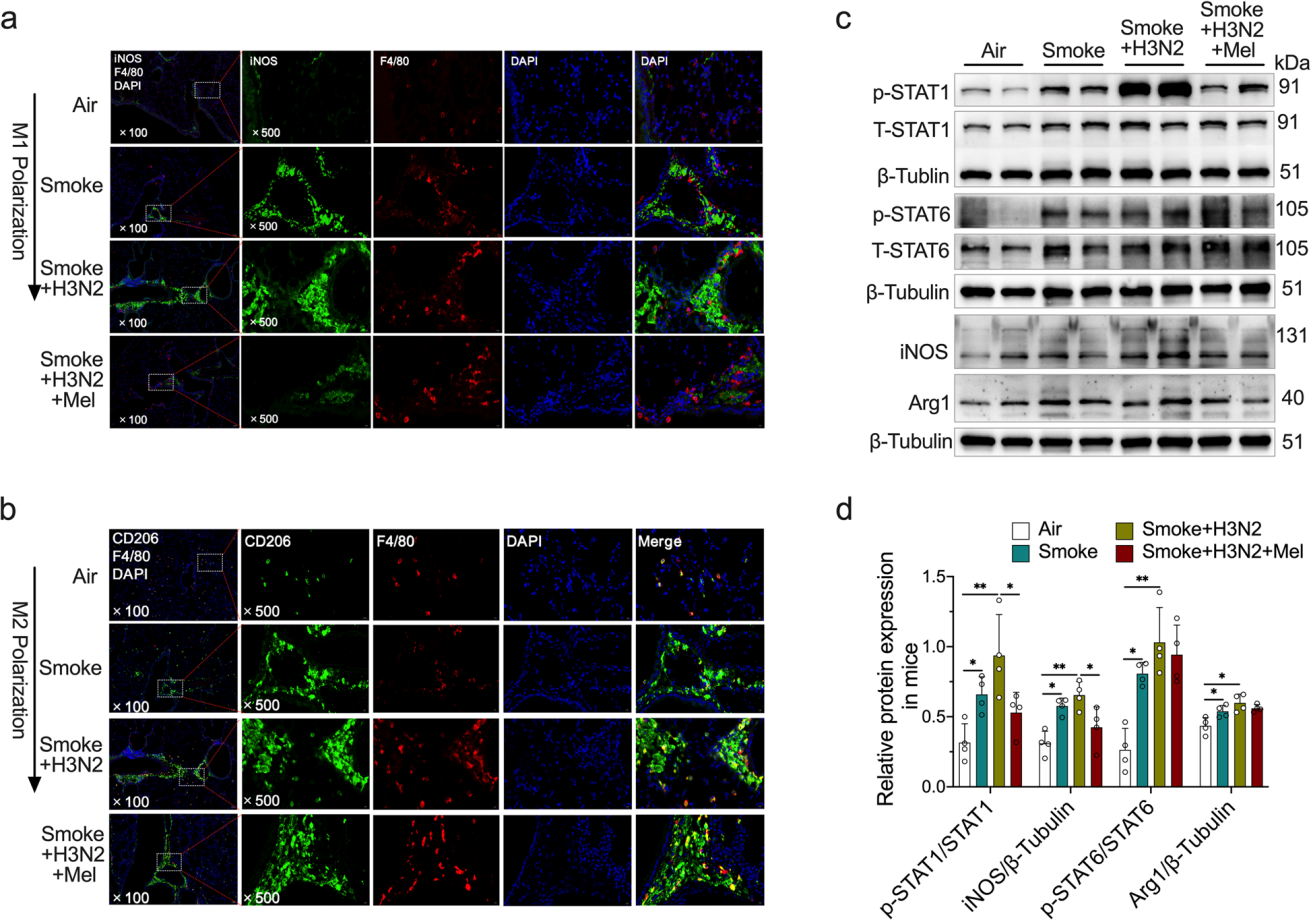
The effects of melatonin on abnormal polarization of pulmonary macrophages in AECOPD. **a**, **b** Mouse lung tissue sections from each group were probed with the specific antibody against pulmonary macrophage marker F4/80 (red) and co-probed with antibodies against M1 marker (iNOS) or M2 marker (CD206), and, representative lung immunofluorescence staining of lung tissue sections were shown (original magnification × 100 and × 500). **c**, **d** Western blot analysis of the expression of total-STAT1, phospho-STAT1, total-STAT6, phospho-STAT6, iNOS and Arg1 to β-tublin in lung tissue homogenates from air group, COPD (CS exposure) group, AECOPD (smoke + H3N2) group, AECOPD + melatonin (Mel, 30 mg/kg) group. Data expressed as mean ± SD (*n* ≥ 3). ^*^p < 0.05, ^**^p < 0.01, ^***^p < *0.001*

In order to investigate the effects of CS exposure plus IAV/H3N2 infection on the polarization of pulmonary macrophages, alveolar macrophages (AMs) in BALF were defined by flow cytometry analysis based on the specific markers of M1 AMs (CD45^+^Siglec-F^+^CD11c^+^CD86^+^population) and M2 AMs (CD45^+^Siglec-F^+^CD11c^+^CD206^+^ population). The results revealed that CS exposure significantly increased the percentage of AMs (Siglec-F^+^CD11c^+^ population) (Fig. 3a-b). Particularly, CD86^+^ (M1 type), CD206^+^ (M2 type) and CD86^+^CD206^+^ AMs were all increased in CS-exposed mice (Fig. 3a, c and e), indicated that CS exposure induced the dual polarization of alveolar macrophages. H3N2 infection further increased the percentages of CD86^+^ AMs and CD86^+^CD206^+^ AMs (Fig. 3a, c and e), indicating that H3N2 infection further promoted dual polarization of AMs in CS-exposed mice. Additionally, immunofluorescence staining of lung tissue sections revealed that the fluorescence intensities of iNOS (M1 type) and CD206 (M2 type) were relatively up-regulated, indicating the dual polarization of pulmonary macrophages in CS-exposed mice (Fig. 4a-b). H3N2 infection further increased iNOS fluorescence intensity, and, CD206 fluorescence intensity also showed an elevated trend compared to CS-exposed mice (Fig. 4a-b). Additionally, western blot analysis of lung tissues also demonstrated that CS exposure up-regulated the phosphorylated levels of STAT1 and STAT6 (Fig. 4c-d), indicating that CS exposure promoted the M1 and M2 polarization of pulmonary macrophages. H3N2 infection further promoted the phosphorylation of STAT1, phosphorylated STAT6 also showed elevated trend after H3N2 infection though had no significance (Fig. 4c-d). These results suggested that IAV infection further promoted the dual polarization of pulmonary macrophages, especially macrophage M1 polarization.

### Melatonin inhibited M1 polarization of pulmonary macrophages in IAV-infected COPD mice

Flow cytometry analysis of BAL cells showed that melatonin treatments decreased the percentage of CD86^+^ AMs and CD86^+^CD206^+^ AMs in IAV/H3N2-infected COPD mice (Fig. 3a-e), indicating the decreased proportion of CD86^+^/CD206^+^ double-positive cells was largely attributed to the decreased expression of CD86 in CD206^+^ AMs. Meanwhile, immunofluorescence staining of lung tissue sections showed melatonin significantly decreased the fluorescence intensity of iNOS without affecting CD206 fluorescence intensity (Fig. 4a-b). Likewise, melatonin inhibited the phosphorylation of STAT1 and iNOS expression, while no effects on the phosphorylation of STAT6 and Arg1 expression in H3N2-infected COPD mice (Fig. 4c-d). These results demonstrated that melatonin inhibited the M1 polarization of pulmonary macrophages in IAV infection-induced AECOPD mice.

### IAV Infection promoted the dual polarization with decreased expression of melatonin receptors in CSE-stimulated Raw264.7 cells

**Fig. 5:**
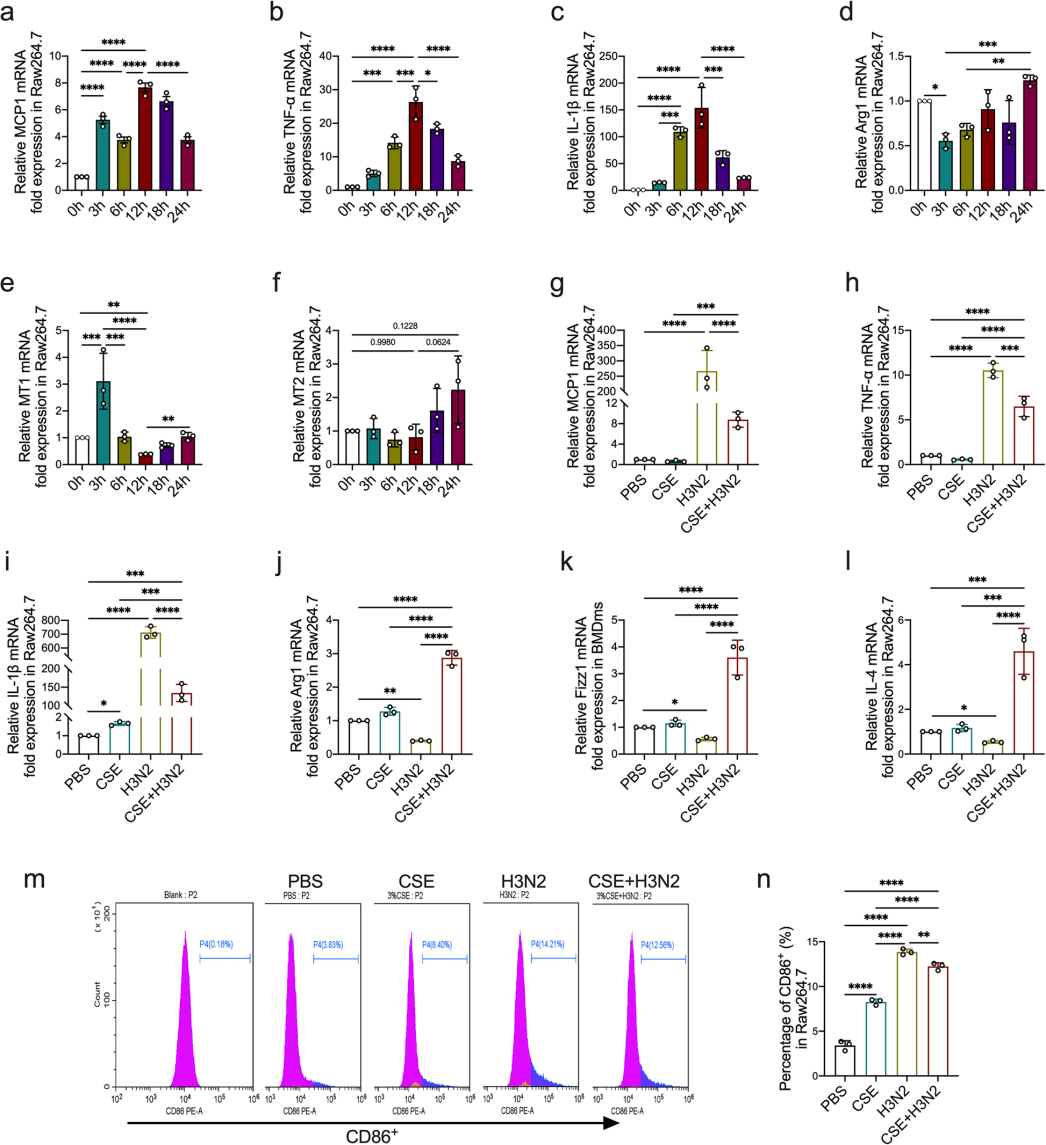
The effects of IAV infection on the polarization of CSE-stimulated Raw264.7 cells. Quantitative reverse transcription-polymerase chain reaction (RT-PCR) measurements of the relative mRNA levels of MCP1 (**a**), TNF-α (**b**), IL-1β (**c**), Arg1 (**d**), melatonin receptor 1 (MT1) (**e**) and MT2 (**f**) in CSE-stimulated Raw264.7 cells infected by influenza A/H3N2 (MOI = 2, infection for 3 h, 6 h, 12 h, 18 h and 24 h). Quantitative RT-PCR measurements of the relative mRNA levels of MCP1 (**g**), TNF-α (**h**), IL-1β (**i**), Arg1 (**j**), fizz1 (**k**) and IL-4 (**l**) in Raw264.7 cells from PBS group, CSE stimulation group, H3N2 infection group, CSE stimulation plus H3N2 infection group. **m**, **n** Flow cytometry analysis to the M1 polarized levels (CD86+) of Raw264.7 cells from each group. Data expressed as mean ± SD (*n* ≥ 3). ^*^p < 0.05, ^**^p < 0.01, ^***^p < 0.001, ^****^p < *0.001*

To simulate IAV/H3N2 infection-induced AECOPD model in vitro, cigarette smoking extract (CSE) was used to pre-stimulate Raw264.7 cells. In CSE-stimulated Raw264.7 cells, H3N2 infection up-regulated the mRNA expression of M1 markers (MCP1, TNF-α and IL-1β) in a time-dependent manner with peak levels observed at 12 h (Fig. 5a-c). Oppositely, MT1 mRNA expression showed a decreased trend from 3 to 12 h, whereas no changes in MT2 mRNA expression (Fig. 5e-f). Upon H3N2 infection, the mRNA expression of M1 markers decreased from 12 to 24 h which coupled with an elevated trend in M2 marker (Arg1), MT1 and MT2 in CSE-stimulated Raw264.7 cells (Fig. 5a-e), indicating that the expression of melatonin receptors were inversely proportional with the expression of M1 markers in IAV-infected CSE-stimulated Raw264.7 cells. Compared to CSE-stimulated Raw264.7 cells, H3N2 infection significantly up-regulated the mRNA expression of M1 markers (MCP1, TNF-α and IL-1β), also increased M2 markers (Arg1, Fizz1 and IL-4) (Fig. 5g-l), showing a dual polarization induced by CSE stimulation combined with IAV infection. However, sole H3N2 infection decreased M2 markers (Fig. 5j-l). Flow cytometry analysis also showed that H3N2 infection further up-regulated the percentage of CD86+ Raw264.7 cells (M1 type) in CSE-stimulated Raw264.7 cells (Fig. 5m-n).

Compared to CSE-stimulated Raw264.7 cells, H3N2 infection significantly up-regulated the mRNA expression of M1 markers (MCP1, TNF-α and IL-1β), also increased M2 markers (Arg1, Fizz1 and IL-4) (Fig. 5g-l), showing a dual polarization induced by CSE stimulation combined with IAV infection. However, sole H3N2 infection decreased M2 markers (Fig. 5j-l). Flow cytometry analysis also showed that H3N2 infection further up-regulated the percentage of CD86^+^ Raw264.7 cells (M1 type) in CSE-stimulated Raw264.7 cells (Fig. 5m-n).

### Melatonin inhibited IAV infection-induced M1 polarization in CSE-stimulated Raw264.7 cells

**Fig. 6:**
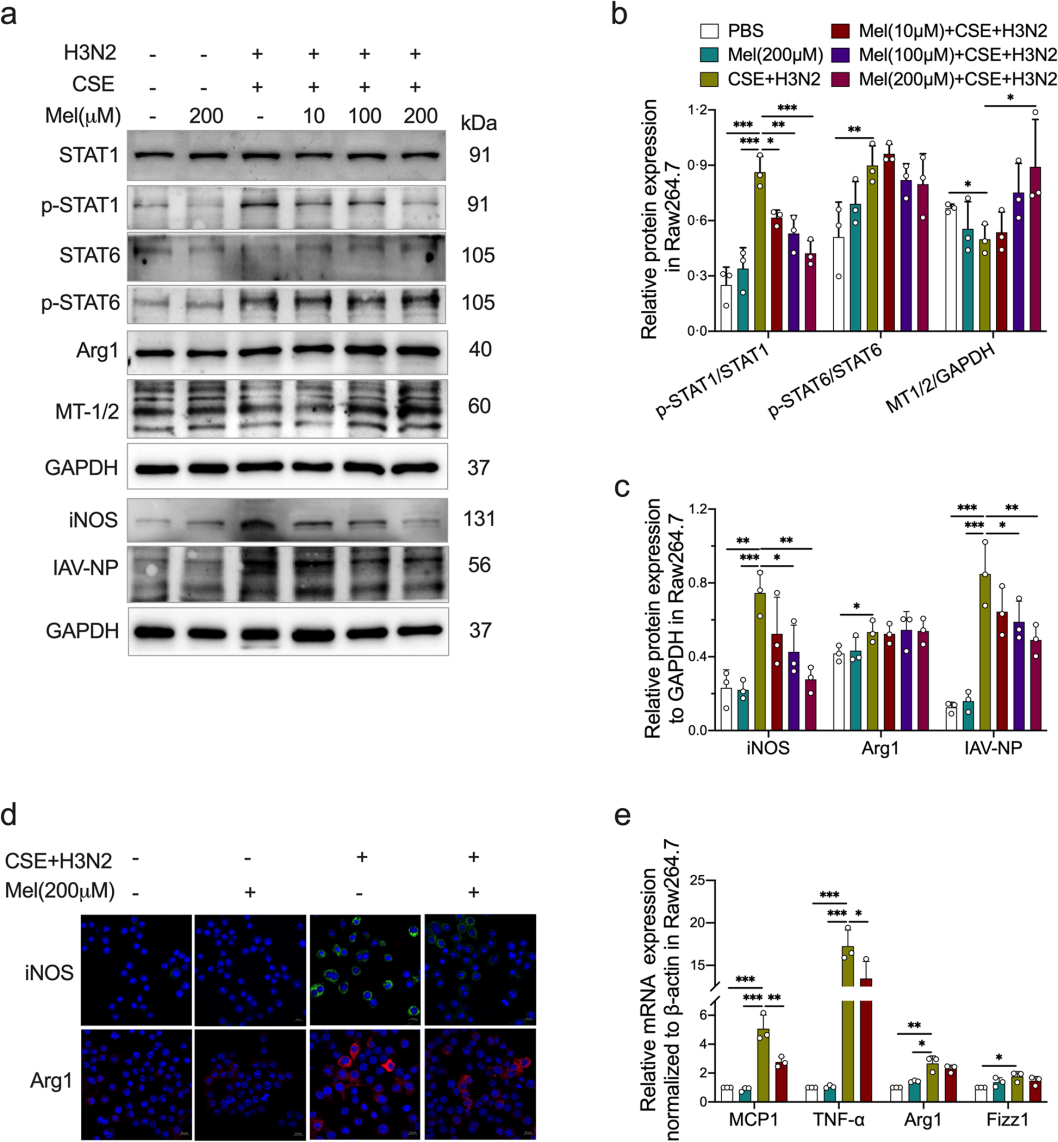
The effects of melatonin abnormal polarization of IAV-infected CSE-stimulated macrophages. **a**-**c** Western blot analysis of the expression of total-STAT1, Phospho-STAT1, total-STAT6, Phospho-STAT6 and IAV-NP to GAPDH as well as iNOS, Arg1 and MT1/2 to GAPDH in CSE-stimulated Raw264.7 cells infected by IAV/H3N2 infection (MOI = 2, 12 h) with/without melatonin pretreatment (10 μM, 100 μM and 200 μM, 3 h before H3N2 infection). **d** Representative Immunofluorescence images of iNOS (green) and Arg1 (red) expression in Raw264.7 cells infected by H3N2 infection (MOI = 2, 12 h) with/without melatonin pretreatment (200 μM, 3 h before H3N2 infection) (bar = 10 μm, original magnification × 630). **e** Quantitative RT-PCR measurements of the relative mRNA levels of MCP1, TNF-α, Arg1 and Fizz1 in CSE-stimulated Raw264.7 cells. Data expressed as mean ± SD (*n* = 3). ^*^p < 0.05, ^**^p < 0.01, ^***^p < 0.001, ^****^p < *0.001*

In CSE-stimulated Raw264.7 cells, there were obvious increases in the phosphorylated levels of STAT1 and STAT6 as well as the protein expression of iNOS and Arg1 after IAV/H3N2 infection (Fig. 6a-c). H3N2 infection also caused an elevation in the levels of IAV-NP protein in CSE-stimulated Raw264.7 cells (Fig. 6a and c). Melatonin treatments inhibited STAT1 phosphorylation and iNOS expression in a dose-dependent manner, but had no inhibitory effects on STAT6 phosphorylation and Arg1 expression (Fig. 6a-c), indicating that melatonin only inhibited the M1 polarization without affecting M2 polarization in H3N2-infected CSE-stimulated Raw264.7 cells. Moreover, melatonin decreased IAV-NP levels with increased MT1/2 expression (Fig. 6a-c). Immunofluorescence staining also showed that melatonin decreased iNOS fluorescence intensity, but had no obvious effect on Arg1 fluorescence intensity in H3N2-infected CSE-stimulated Raw264.7 cells (Fig. 6d). PCR analysis showed that CSE and H3N2 co-stimulation up-regulated the mRNA expression of MCP1 and TNF-α, which were both inhibited by melatonin (Fig. 6e). However, melatonin failed to inhibit CSE and H3N2 co-stimulation-induced increases in the mRNA expression of Arg1 and Fizz1 (Fig. 6e). These results suggested that melatonin improved CSE and IAV co-stimulation-induced macrophage injuries via inhibiting M1 polarization.

### Melatonin inhibited IAV infection-induced M1 polarization in a MT-dependent manner in CSE-stimulated Raw264.7 cells

**Fig. 7:**
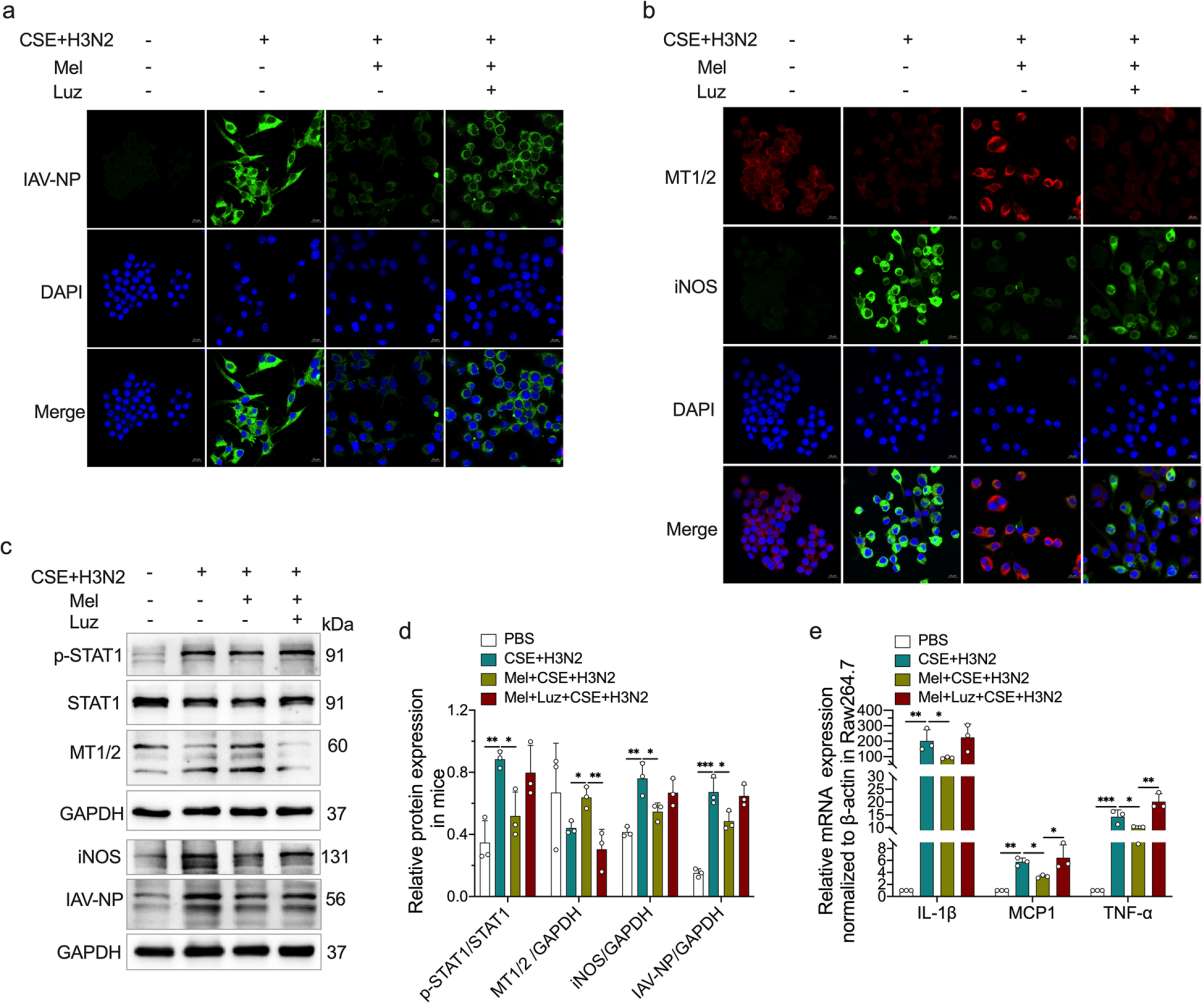
The effects of melatonin receptors on the regulatory impacts of melatonin. Representative Immunofluorescence images of IAV-NP (green) (**a**), iNOS (green) and MT1/2 (red) (**b**) expression in CSE-stimulated Raw264.7 cells infected by influenza A/H3N2 infection (MOI = 2, 12 h) with/without melatonin pretreatment (200 μM) or combined pretreatment of melatonin and luzindole (10 μM, 3 h before H3N2 infection) (bar = 10 μm, original magnification × 630). **c**, **d** Western blot analysis of the expression of total-STAT1, Phospho-STAT1, MT1/2, iNOS and IAV-NP to GAPDH in CSE-stimulated Raw264.7 cells infected by influenza A/H3N2 infection. **e** Quantitative RT-PCR measurements of the relative mRNA levels of IL-1β, MCP1 and TNF-α in CSE-stimulated Raw264.7 cells. Data expressed as mean ± SD (n ≥ 3). ^*^p < 0.05, ^**^p < 0.01, ^***^p < 0.001, ^****^p < *0.001*

To investigate whether the inhibitory impacts of melatonin on macrophage M1 polarization occurred through its receptors, a melatonin receptor antagonist (luzindole), was pretreated combined with melatonin in Raw264.7 cells. Immunofluorescence staining showed that the fluorescence intensity of IAV-NP significantly enhanced in H3N2-infected Raw264.7 cells, which was reduced by melatonin (Fig. 7a). Specially, luzindole alleviated the inhibitory impact of melatonin on IAV-NP expression (Fig. 7a). Immunofluorescence staining also showed that melatonin treatment significantly enhanced MT1/2 fluorescence intensity coupled with reduced iNOS fluorescence intensity (Fig. 7b). However, luzindole reduced the inhibitory impacts of melatonin on iNOS via inhibiting MT1/2 expression (Fig. 7b). The above results indicated that MT1/2 fluorescence intensity was inversely correlated with iNOS and IAV-NP.

In western blot analysis, the inhibitory effects of melatonin on STAT1 phosphorylation and the protein expression of iNOS and IAV-NP were reduced after luzindole addition in CSE and H3N2 co-stimulated Raw264.7 cells (Fig. 7c-d). PCR analysis showed that melatonin failed to down-regulate the mRNA expression of IL-1β, MCP1 and TNF-α after luzindole addition in CSE and H3N2 co-stimulated Raw264.7 cells (Fig. 7e). Collectively, these results demonstrated that melatonin relied on melatonin receptors to inhibit macrophage M1 polarization.

### IL-1β abolished the inhibitory impacts of melatonin on M1 polarization via strengthening STAT1 phosphorylation

**Fig. 8:**
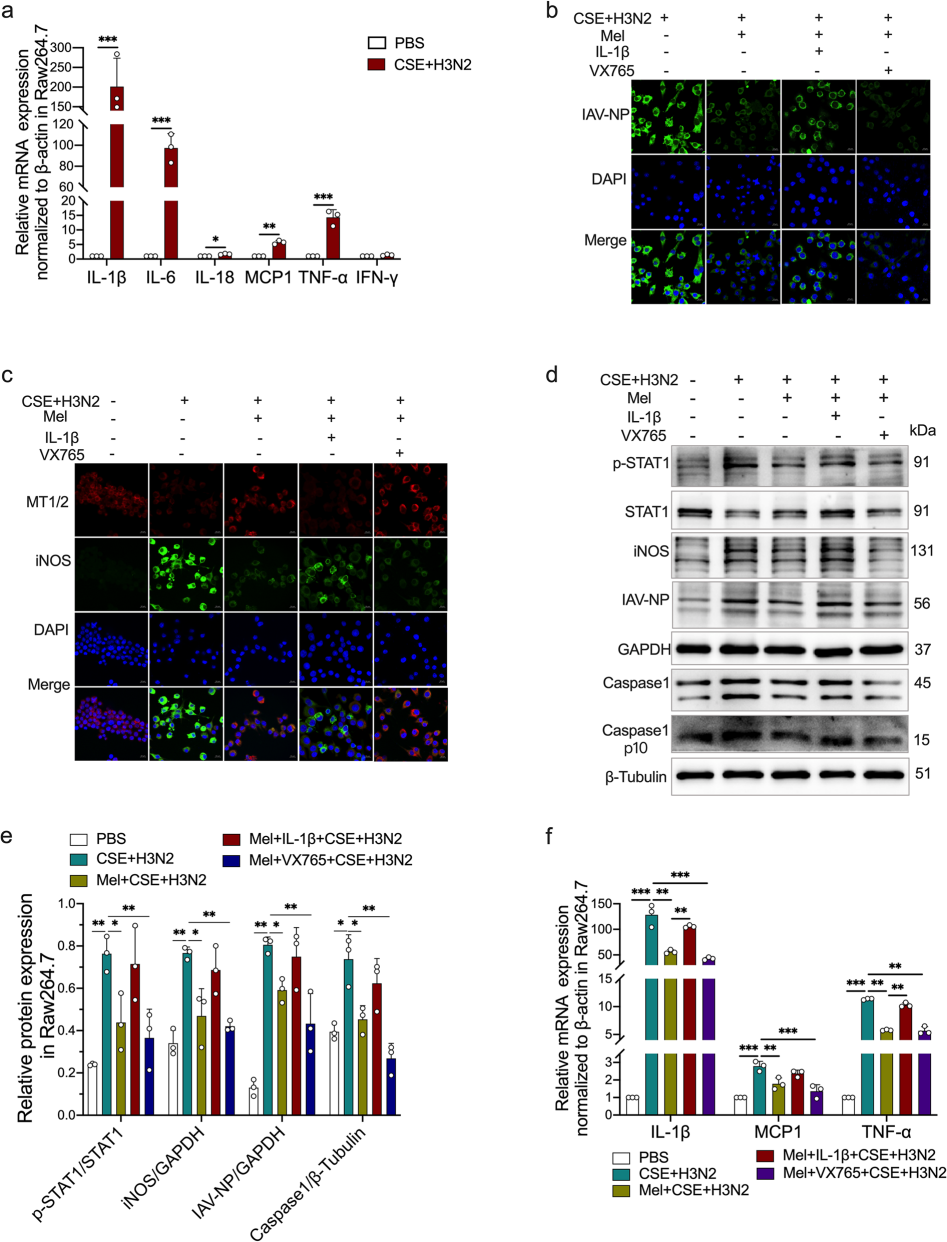
The effects of IL-1β on the regulatory impacts of melatonin. **a** Quantitative RT-PCR measurements of the relative mRNA levels of IL-1β, IL-6, IL-18, MCP1, TNF-α and IFN-γ in CSE-stimulated Raw264.7 cells infected by IAV/H3N2 infection (MOI = 2, 12 h). Representative Immunofluorescence images of IAV-NP (green) (**b**), iNOS (green) and MT1/2 (red) (**c**) expression in CSE-stimulated Raw264.7 cells infected by H3N2 infection (MOI = 2, 12 h) with/without melatonin pretreatment (200 μM) or combined pretreatment of melatonin and re-IL-1β (10 μg/ml) or VX765 (50 μM) (bar = 10 μm, original magnification × 630). **d**, **e** Western blot analysis of the expression of total-STAT1, phospho-STAT1, iNOS and IAV-NP to GAPDH as well as Caspase1 and Caspase1 p10 to β-Tublin in Raw264.7 cells. **f** Quantitative RT-PCR measurements of the relative mRNA levels of MCP1, TNF-α and IL-1β in Raw264.7 cells. Data expressed as mean ± SD (*n* = 3). ^*^p < 0.05, ^**^p < 0.01, ^***^p < 0.001, ^****^p < *0.001*

In CSE-stimulated Raw264.7 cells, IAV/H3N2 infection further promoted the expression of inflammatory factors, including IL-1β, IL-6, IL-18, MCP1, TNF-α and IFN-γ. Therein, IL-1β mRNA expression was the highest one, increased approximate 200 times compared to that of PBS-treated cells (Fig. 8a). This finding suggested that the burst elevation of IL-1β contributed to IAV-driven further activation of M1 polarization in CSE-stimulated macrophages.

Next, immunofluorescence staining of Raw264.7 cells showed that re-IL-1β addition increased IAV-NP fluorescence intensity, while VX765 further decreased it compared to single melatonin-treated Raw264.7 cells (Fig. 8b). And, re-IL-1β addition decreased MT1/2 fluorescence intensity, but enhanced the fluorescence intensity of iNOS (Fig. 8c). However, VX765 addition further enhanced MT1/2 fluorescence intensity, decreased that of iNOS (Fig. 8c). Compared to single melatonin intervention, re-IL-1β abolished while VX765 enhanced the inhibitory effects of melatonin on the phosphorylation of STAT1 and the protein expression of iNOS, IAV-NP and Caspase1 (Fig. 8d-e), as well as the mRNA expression of MCP1, TNF-α and IL-1β (Fig. 8f). These results suggested that IL-1β contributed to STAT1-driven M1 polarization in CSE and H3N2 co-stimulated Raw264.7 cells. Since treatments with either luzindole or IL-1β abolished the inhibitory effect of melatonin on STAT1 phosphorylation, we considered that melatonin suppressed H3N2 infection-induced M1 polarization via inhibiting IL-1β/STAT1 signaling in MTs-dependent manners in CSE-stimulated macrophages.

### Melatonin reduced CSE and IAV co-stimulation-induced apoptosis by suppressing IL-1β/STAT1 signaling

**Fig. 9:**
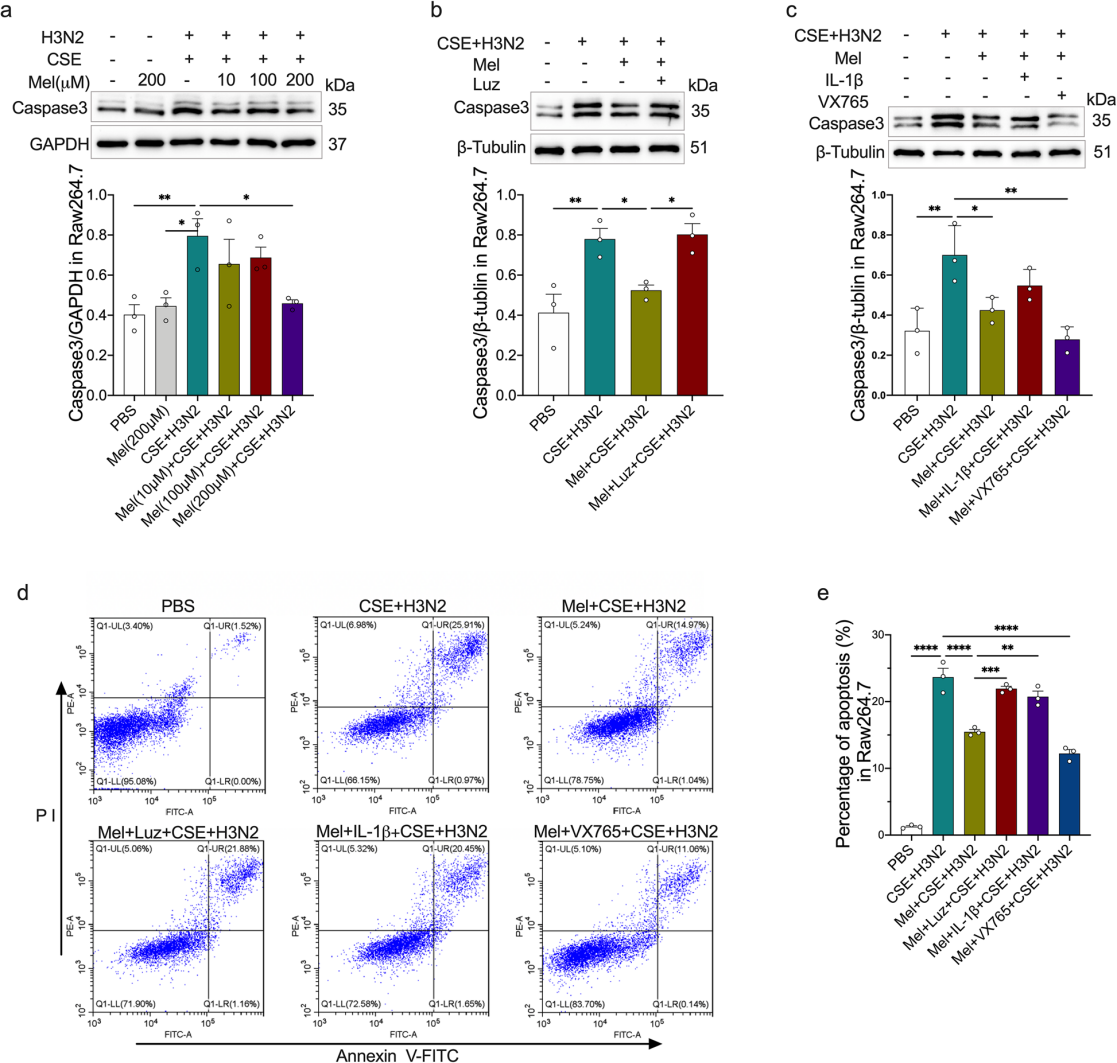
The effects of melatonin on macrophage apoptosis. **a** Western blot analysis of the expression of Caspase3 to GAPDH in CSE-stimulated Raw264.7 cells infected by IAV/H3N2 (MOI = 2, 12 h) with/without melatonin pretreatment (10 μM, 100 μM and 200 μM, 3 h before H3N2 infection). **b**, **c** Western blot analysis of the expression of Caspase3 to β-Tublin in CSE-stimulated Raw264.7 cells infected by H3N2 infection with/without melatonin pretreatment (200 μM) or combined pretreatments of melatonin and luzindole (10 μM) or re-IL-1β (10 μg/ml) or VX765 (50 μM). **d**, **e** Flow cytometry analysis of Annexin V^+^ and Propidium Iodide (PI^+^) was done to identify apoptosis in Raw264.7 cells. Data expressed as mean ± SD (*n* = 3). ^*^p < 0.05, ^**^p < 0.01, ^***^p < 0.001, ^****^p < *0.001*

Finally, we investigated the anti-apoptotic ability of melatonin and its potential mechanisms. Western blot analysis showed that CSE and H3N2 co-stimulation increased Caspase3 expression, while melatonin inhibited such increase in a dose-dependent manner (Fig. 9a). After luzindole addition, the inhibitory effects of melatonin decreased (Fig. 9b). Likewise, re-IL-1β addition reduced while VX765 enhanced the inhibitory effect of melatonin on Caspase3 expression (Fig. 9c). Furthermore, melatonin decreased the percentages of apoptosis in H3N2-infected CSE-stimulated Raw264.7 cells. The addition of luzindole or re-IL-1β elevated the percentages of apoptosis cells compared to that of single melatonin treatment, while VX765 further decreased the percentage of apoptosis cells (Fig. 9d-e), indicating that IL-1β levels affected the anti-apoptotic ability of melatonin, which exerted anti-apoptotic effects probably in a MT-dependent manner.

## Discussion

Long-term CS exposure leads to pulmonary toxin accumulation, elevates pro-inflammatory and pro-apoptotic factors, ultimately causes COPD. Bacteria and viruses infections are easier to induce the exacerbation of COPD symptoms [34]. Our previous study indicated that influenza viruses were the most common respiratory viruses for AECOPD in Asia [5]. Particularly, macrophage polarization is the main cause driving cell “injuries-repairments” and apoptosis in lung tissues of COPD [14, 35]. Melatonin is a well-recognized neurohormone with anti-inflammatory and anti-apoptotic functions. In this study, we clearly verified that melatonin inhibited influenza A virus (IAV) infection, improved lung function, protected against IAV infection-induced lung damages in cigarette smoking-induced COPD mice. Mechanistically, melatonin inhibited macrophage M1 polarization and apoptosis by suppressing IL-1β/STAT1 signaling in MTs-dependent manners.

Cumulative evidence suggested that macrophages were obviously increased in lung microenvironments of COPD patients, mainly including AMs and lung interstitial macrophages (IMs) [36]. Therein, AMs are the first-line defenders of airway and alveoli immunoregulation, IMs are the gatekeepers of lung microvasculatures and parenchyma [37]. Long-term cigarette irritation causes recruitments of circulating monocytes into lung, subsequently differentiates into AMs. Previous studies showed that M1 and M2 phenotype macrophages largely existed in BALF of COPD patients [38]. Abnormal polarized AMs are the main triggers of airway cytokine storms [39]. In COPD, pathogens infection may further evoke abnormal polarization of AMs, exacerbate lung damages of COPD patients [15, 40]. In our previous study, acute IAV infection was found only up-regulated the M1 polarization of pulmonary macrophages [18]. We speculate that, while macrophages are in resting status, acute IAV infection dominantly polarizes them into pro-inflammatory M1 phenotype. However, on the basis of cigarette stimulation, macrophages are not in resting status, they has been activated in both pro-inflammatory (M1) and pro-proliferative (M2) status [12, 41]. On such activated basis, additional acute IAV infection initially induces M1 polarization to produce pro-inflammatory intermediators and induces acute inflammatory infiltration. Contrarily, with IAV infection prolonging, to counterbalance the infiltrated inflammation, M2 macrophages is activated to defend from IAV-induced injury and trigger wound healing [14, 15, 42]. As reflected in Fig. 5, in CSE-stimulated macrophages, M1 markers (MCP1 and IL-1β) were elevated prior to M2 marker (Arg1) in response to IAV infection.

Enormous studies indicated that melatonin had potent protective effects on lung diseases induced by bacteria, influenza virus and SARS-CoV2 infection [23, 27, 28]. Particularly, melatonin reduces the viral entry and viral replication, inhibits systemic inflammation by suppressing NF-κB signaling and the expression of iNOS and COX2 (M1 markers) [23]. Consistently, our results demonstrated that melatonin inhibited IAV infection as shown by decreased IAV-NP expression, and improved lung damages of AECOPD mice. And, its protective mechanisms may attribute to the regulation of macrophage polarization. Since phosphorylated STAT1 moves into the nucleus, regulates M1 polarization, whereas the regulation of M2 polarization primarily relies on STAT6 signaling [43, 44]. In our results, melatonin significantly inhibited macrophage M1 polarization via inhibiting STAT1 phosphorylation in vivo and in vitro, which was in agreements with our previous study in ALI induced by H3N2 infection [18]. However, melatonin had no significant impacts on STAT6 phosphorylation after IAV infection in cigarette-stimulated microenvironments, which is conversed with single H3N2-infected micro-environments [18]. This distinction may be due to abnormal pulmonary inflammatory microenvironments with highly activated M1 and M2 AMs induced by chronic cigarette irritation [12, 41]. Specific mechanisms remains to be studied.

And, melatonin receptors (MTs) are involved in the regulatory impacts of melatonin. CSE and H3N2 co-stimulation decreased MT1 expression, but no significant change in MT2 expression. Moreover, blocking MTs by luzindole attenuated the inhibitory effects of melatonin on macrophage M1 polarization and resistant of IAV, indicating that an intact “melatonin-MTs” axis on the membrane is requisite for the impacts of melatonin on anti-IAV infection and inhibiting macrophage M1 polarization. Likewise, a previous study demonstrated that blocking of MT significantly weakened the anti-apoptotic and anti-oxidative capacities of melatonin [33]. Commonly, MT2 is mainly expressed in the brain, MT1 is widely expressed in the brain, peripheral organs and immune cells [45], which indicates that MT1 may be the main membrane binding receptor for melatonin in the peripheral including lung tissues. Blocking MT1 may be a potential direction for anti-inflammation and anti-IAV infection in AECOPD.

As a key immune regulatory factor, high-level IL-1β is a notable feature of severe influenza virus or SARS-CoV2 infection [46, 47]. As expected, IAV infection up-regulated IL-1β mRNA expression approximately 200 times, which was far more than IL-6, IL-18, MCP1, TNF-α and IFN-γ, indicating that the important pro-inflammatory role of IL-1β in IAV-infected CSE-stimulated macrophages. IL-1β is mainly expressed by M1-type macrophages, its mature form derives from the cleavage of activated Caspase1 to pro-IL-1β [48]. IL-1β promotes the transcriptional expression of IL-6, which further recruits neutrophils into lung [49]. IL-1β can also promote STAT1 phosphorylation, induce the expression of iNOS, MCP1, CXCL1/2 and IL-6 [50, 51]. In this study, re-IL-1β decreased the inhibitory effects of melatonin on STAT1-driven macrophage M1 polarization, and this adverse effects were reversed by IL-1β-converting enzyme inhibitor VX765. IL-1β may plays an joint role between melatonin and STAT1 signaling, indicating that the rationality of “Melatonin-IL-1β/STAT1 signaling axis”.

Apoptosis is also a extremely distinctive feature in COPD, closely associated with lung damages and emphysema [52]. Cigarette smoke contains multiple toxic substances which cause DNA damages and the activation of pro-inflammatory and pro-apoptotic signals [53]. Macrophage M1 polarization can accelerate apoptosis via activating NF-κB or STAT1 signaling [54, 55]. In this study, cigarette irritation induced macrophage apoptosis coupled with the activation of STAT1 signaling. IAV infection further increased apoptosis with enhanced STAT1 phosphorylation. This results suggested that IAV infection may exacerbate lung damages of COPD more rapidly and violently in the chronic impaired pulmonary microenvironments induced by long-term CS exposure. Melatonin has been shown to inhibit apoptosis in a SIRT1-dependent manner [33]. Similarly, we verified that melatonin inhibited apoptosis with the suppression of STAT1 signaling. And, blocking MTs decreased the anti-apoptotic ability of melatonin, indicating that melatonin exerted the anti-apoptotic impact in a MTs-dependent manner. Moreover, re-IL-1β decreased while VX765 enhanced the anti-apoptotic impacts of melatonin, reflecting that IL-1β levels affected the anti-apoptotic impacts of melatonin. Collectively, we proposed that melatonin inhibited apoptosis via suppressing IL-1β/STAT1 signaling in a MTs-dependent manner.

## Conclusions

We provided highly persuasive evidence that IAV infection further promoted the dual polarization and apoptosis of macrophages upon cigarette irritation, and accelerated the progression of COPD. And, melatonin inhibited IAV infection, improved lung function, ultimately alleviated lung damages of AECOPD mice. Particularly, melatonin inhibited macrophage M1 polarization and apoptosis via suppressing IL-1β/STAT1 signaling in a MTs-dependent manner. These findings suggested that melatonin may be a potent agent for improving influenza virus infection-induced AECOPD, and provided potential signaling targets for the studies of the therapeutic strategies of COPD or AECOPD.

### Supplementary Information

**Additional file 1.** Primer sequences used for RT-PCR analysis.

**Additional file 2.**

## Data Availability

No datasets were generated or analysed during the current study.

## Abbreviations

IAV: Influenza A virus
COPD: Chronic obstructive pulmonary disease;
IAV-NP: IAV nucleoprotein
CSE: Cigarette smoke extracts
MT: Melatonin receptor
WHO: World Health Organization
STAT1: Signal transducer and activator of transcription 1
MCP1: Monocyte chemoattractant protein 1
iNOS: Inducible nitric oxide synthase
Arg1: Arginase 1
AMs: Alveolar macrophages
STAT6: Signal transducer and activator of transcription 6
TNF-α: Tumor Necrosis Factor α
Fizz1: Found in inflammatory zone 1
IFN-γ: Interferon γ
ALI: Acute lung injury
ER: Endoplasmic reticulum
SARS-CoV-2: Severe acute respiratory syndrome coronavirus 2
MDCK: Madin-Darby canine kidney
Mel: Melatonin
H&E: Haematoxylin and eosin
BALF: Bronchoalveolar lavage fluid
RBC: Red blood cell
MOI: Multiplicity of infection
TSA: Tyramide signal amplification
Lm: Alveolar mean linear intercept
IMs: Interstitial macrophages
