# Supplementary material for: Melatonin improves influenza virus infection-induced acute exacerbation of COPD by suppressing macrophage M1 polarization and apoptosis
Source: Respir Res. 2024 Apr 27;25:186. doi: 10.1186/s12931-024-02815-0 (PMC11056066; doi:10.1186/s12931-024-02815-0)
Supplement: Supplementary file 1 — Additional file 1. Primer sequences used for RT-PCR analysis. [file 12931_2024_2815_MOESM1_ESM.docx]

**Primer sequences used for RT-PCR analysis**

| **Gene** | **Forward Primer** | **Reverse Primer** |
| --- | --- | --- |
| Mouse-β-actin | CATTGCTGACAGGATGCAGAAGG | TGCTGGAAGGTGGACAGTGAGG |
| Mouse-IL-1β | TGGACCTTCCAGGATGAGGACA | GTTCATCTCGGAGCCTGTAGTG |
| Mouse-TNF-α | GGTGCCTATGTCTCAGCCTCTT | GCCATAGAACTGATGAGAGGGAG |
| Mouse-IL-6 | TCCAGTTGCCTTCTTGGGAC | GTACTCCAGAAGACCAGAGG |
| Mouse-IL-18 | AGGGTTTGTGTTCCAGAAAGATG | AGCCTCGGGTATTCTGTTATGG |
| Mouse-IFN-γ | CTGGAGGAACTGGCAAAAGGATGG | GACGCTTATGTTGTTGCTGATGGC |
| Mouse-MCP1 | CCAATGAGTAGGCTGGAGAGCT | GTCTGGACCCATTCCTTCTTGG |
| Mouse-Arg1 | ACAAGACAGGGCTCCTTTCA | AGCAAGCCAAGGTTAAAGCC |
| Mouse-Fizz1 | ATCGTGGAGAATAAGGTCAAGG | TTGACACTAGTGCAAGAGAGAG |
| Mouse-MT1 | GGAGGGTGAAACCTGACGAC | CCCAGCAAATGGCAAAGAGG |
| Mouse-MT2 | GGCTCCGTCTTCAACATCACC | GCAGAAGGACCAGCAGGGTG |
