# Supplementary material for: Melatonin improves influenza virus infection-induced acute exacerbation of COPD by suppressing macrophage M1 polarization and apoptosis
Source: Respir Res. 2024 Apr 27;25:186. doi: 10.1186/s12931-024-02815-0 (PMC11056066; doi:10.1186/s12931-024-02815-0)

Original full-length gels of Fig. 2f

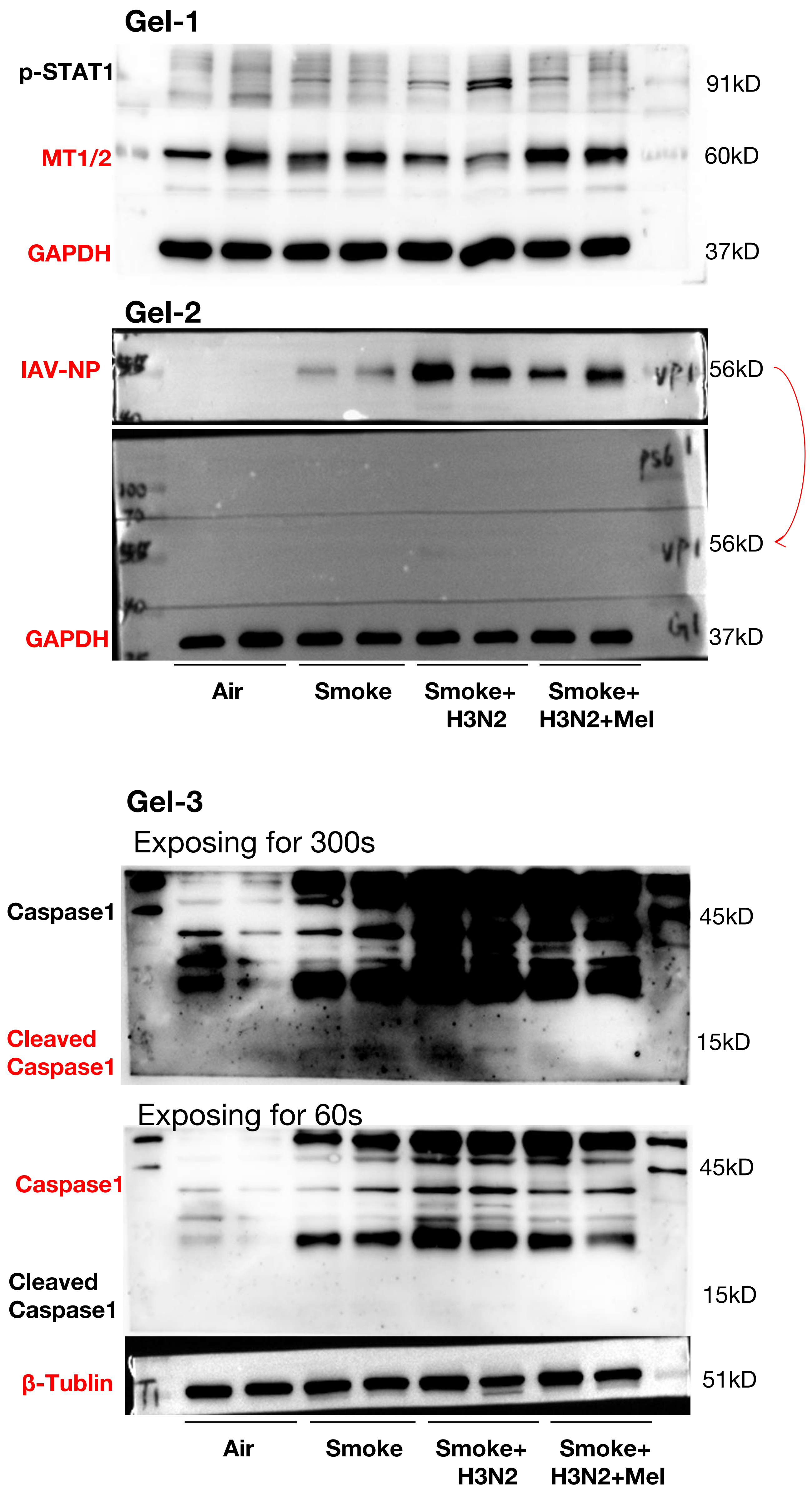

Original full-length gels of Fig. 4c

Gel-1

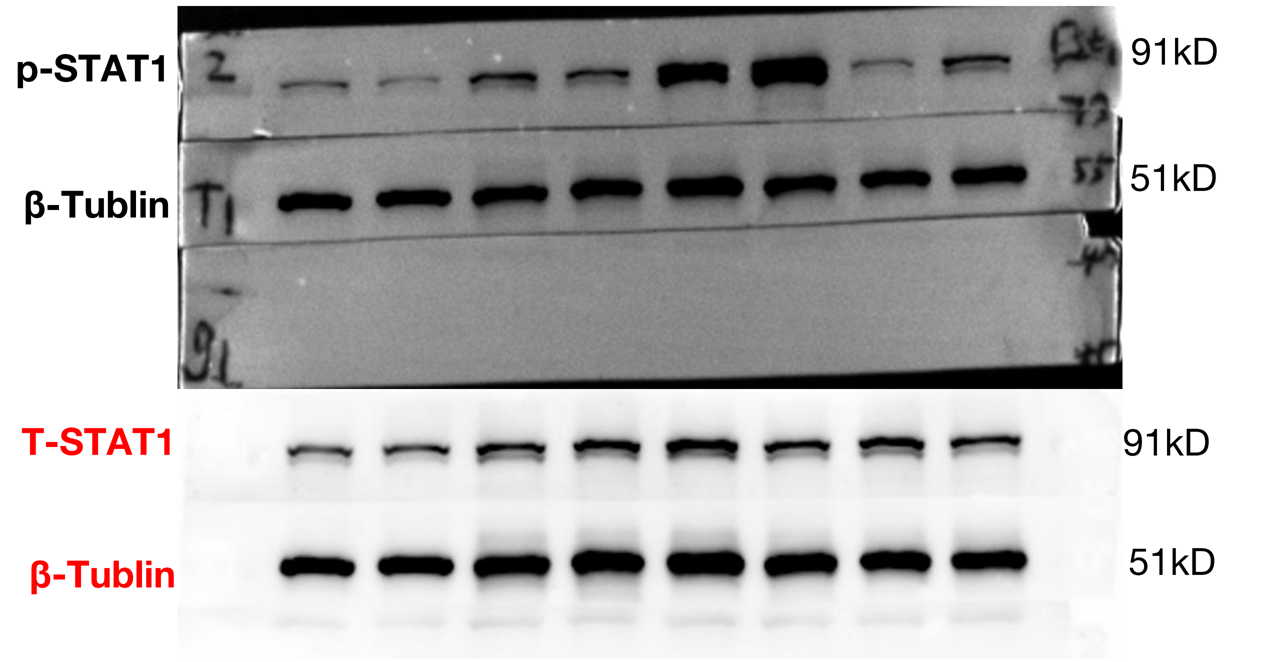

Gel-2

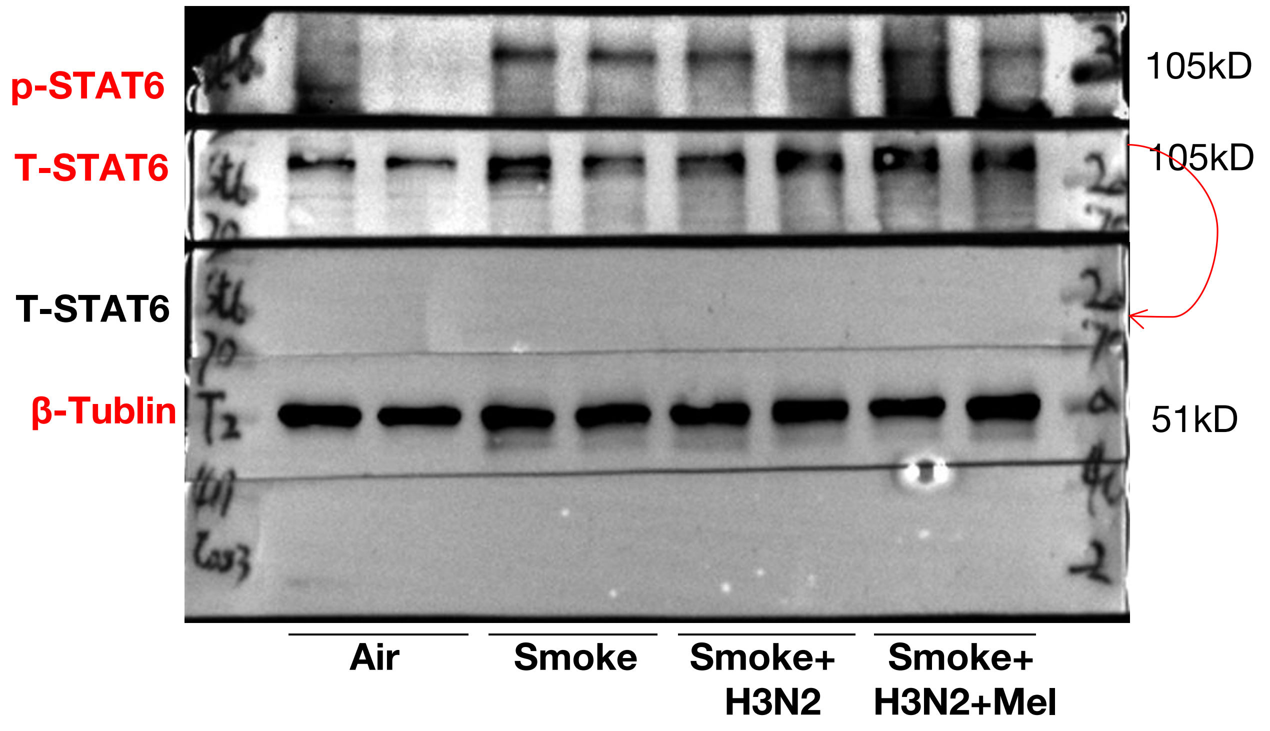

Gel-3

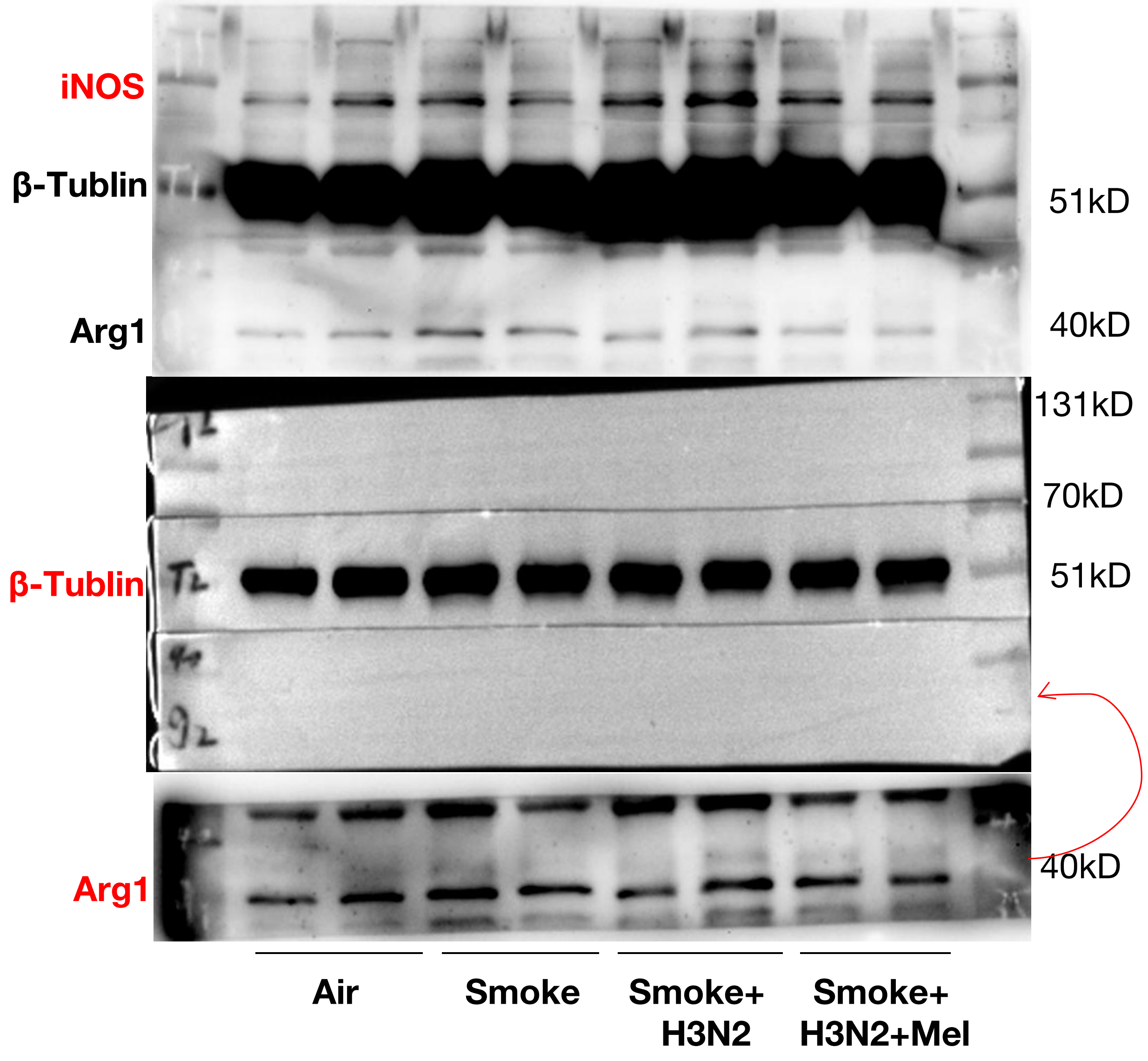

Original full-length gels of Fig. 6a + Fig. 9a

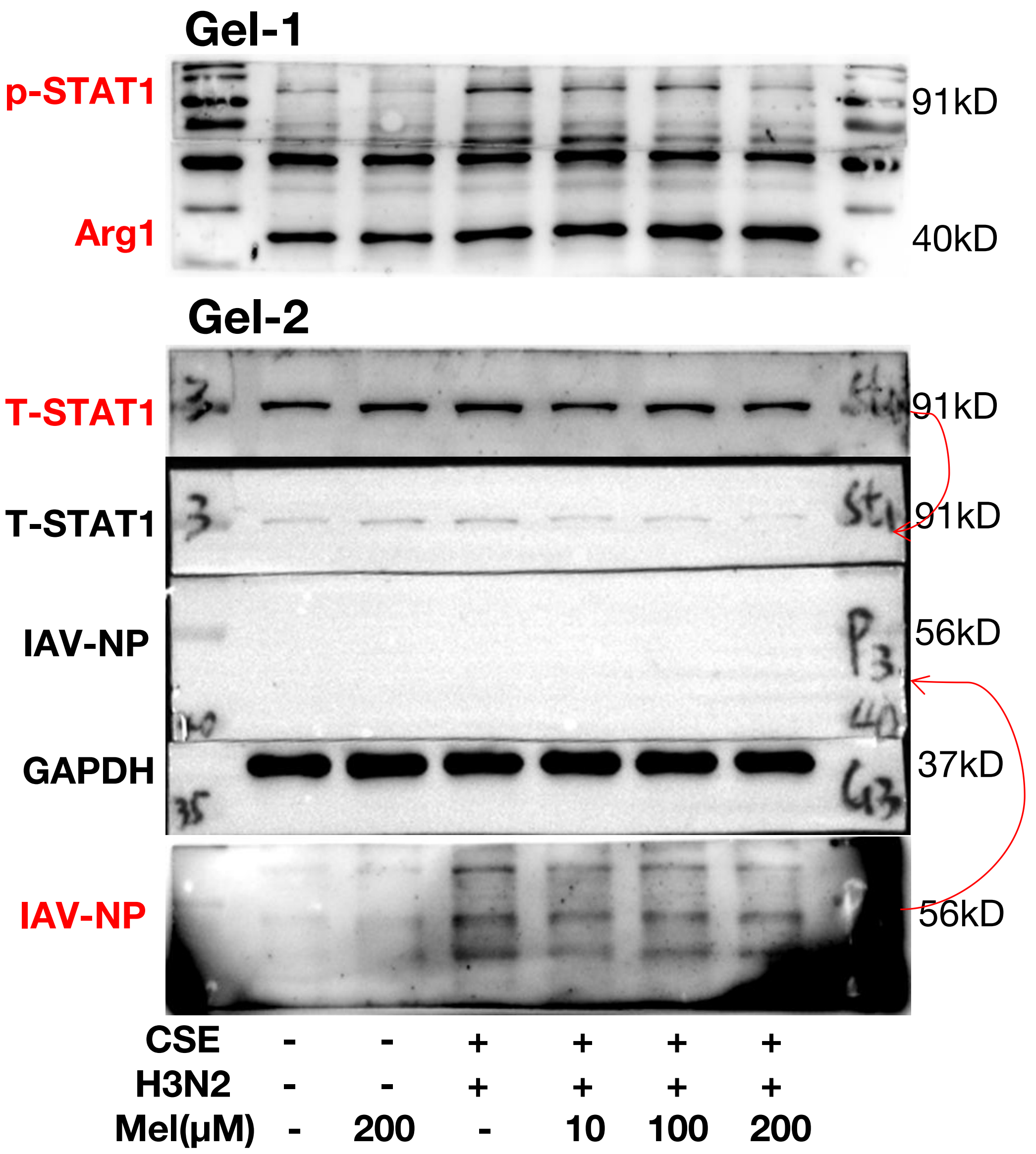

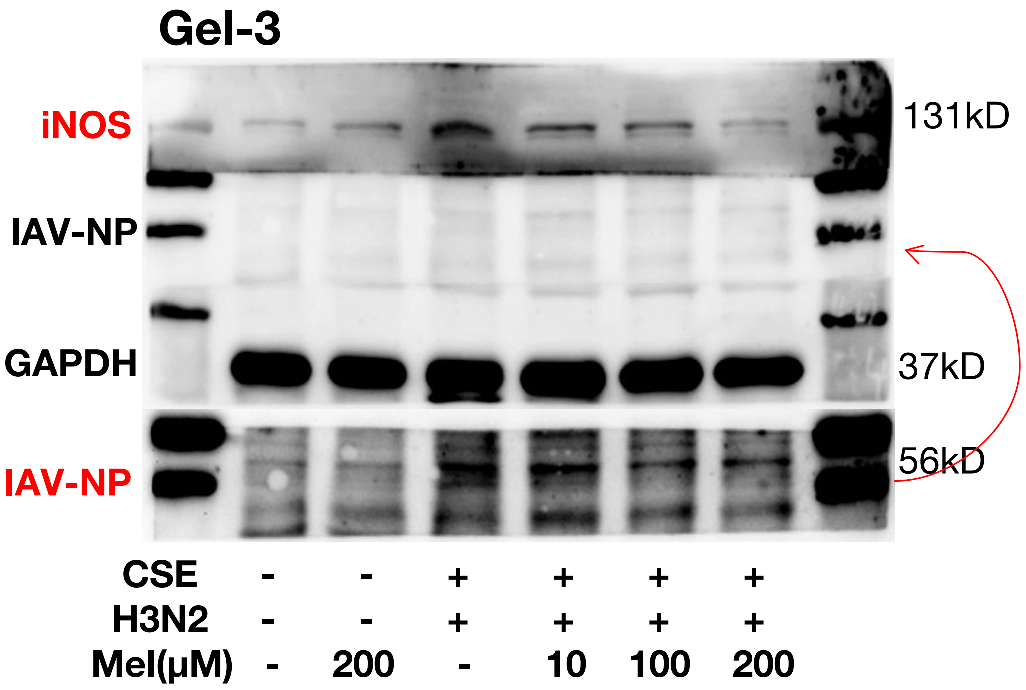

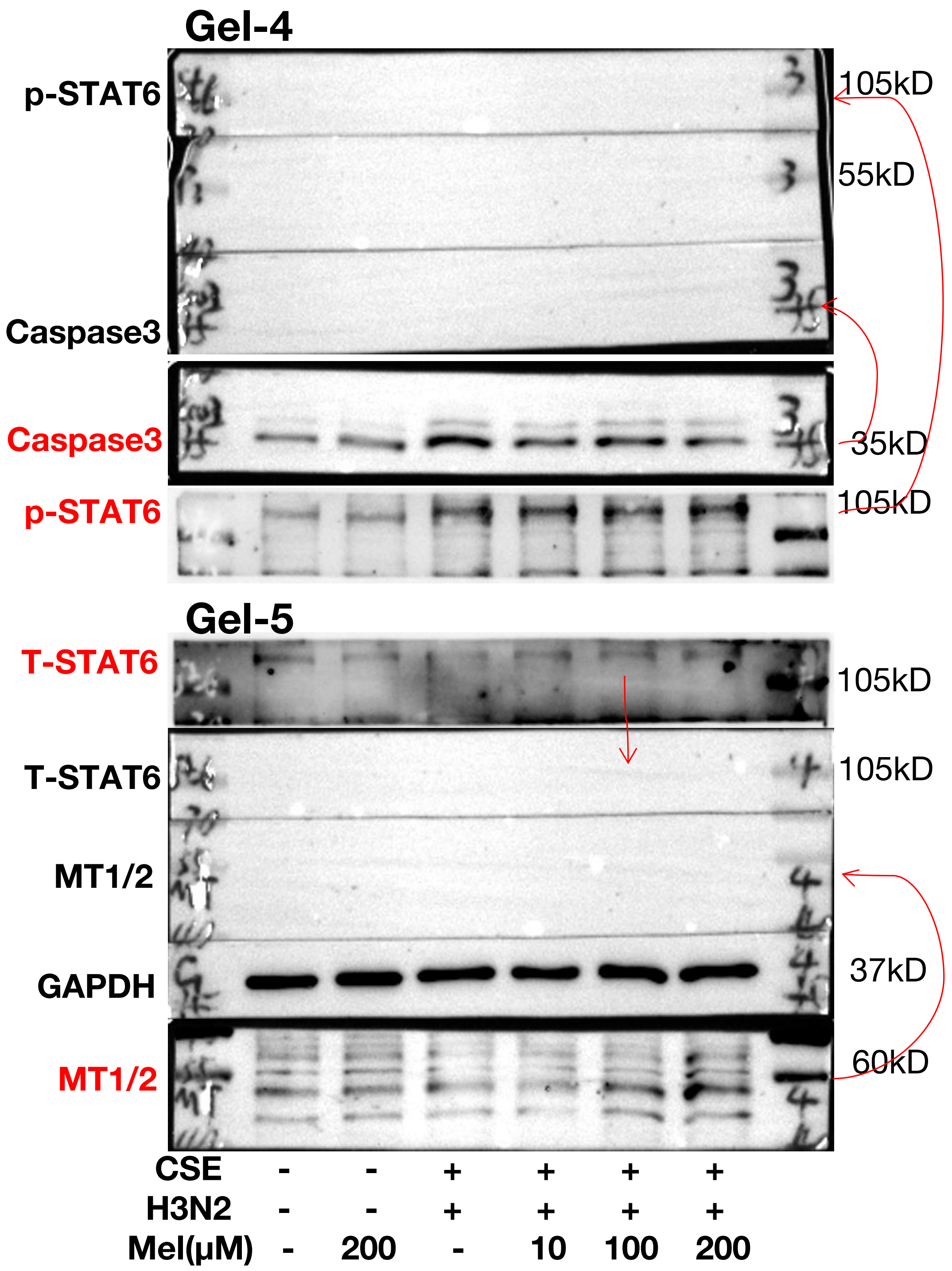

Original full-length gels of Fig. 7c + Fig. 9b

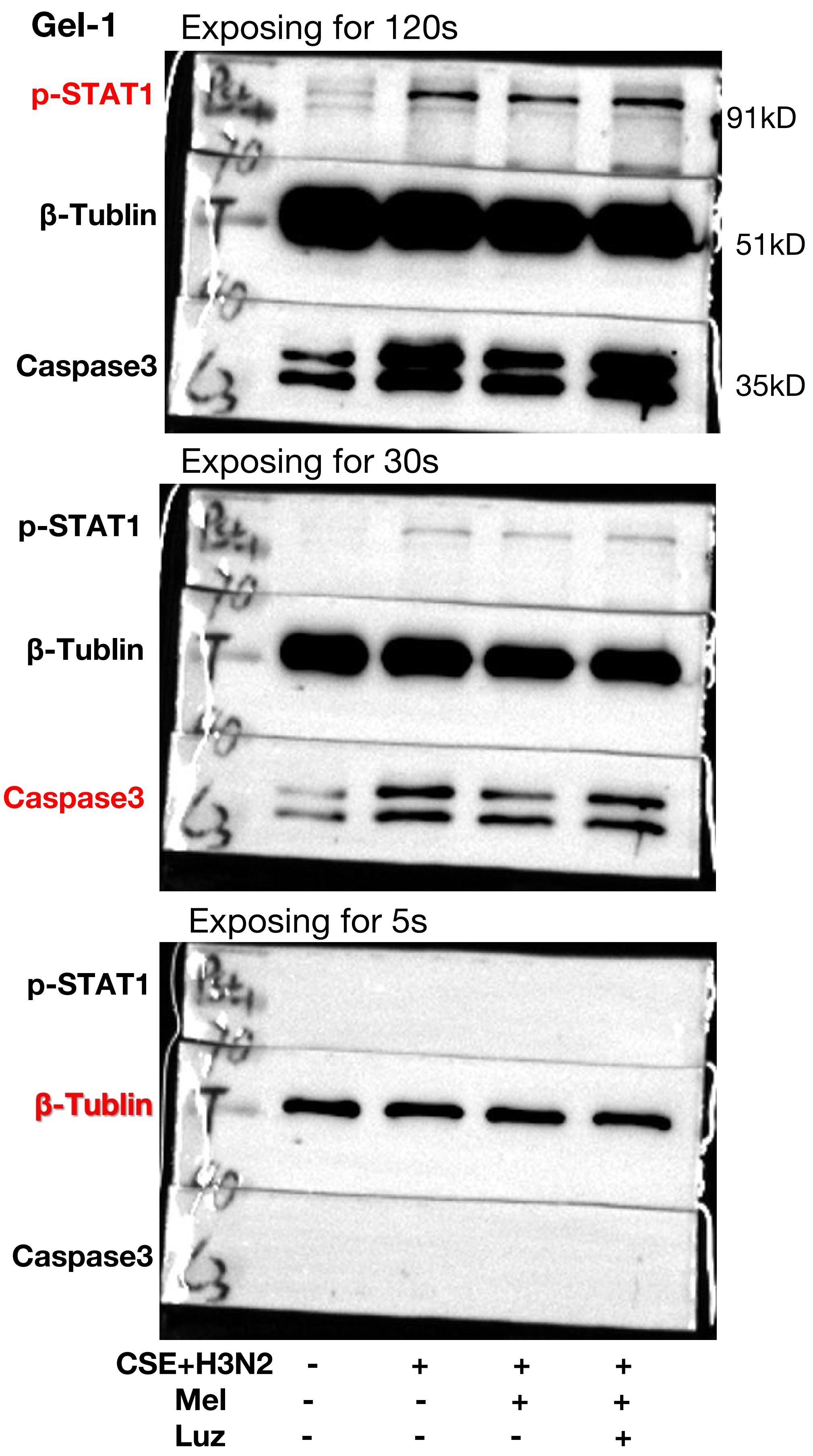

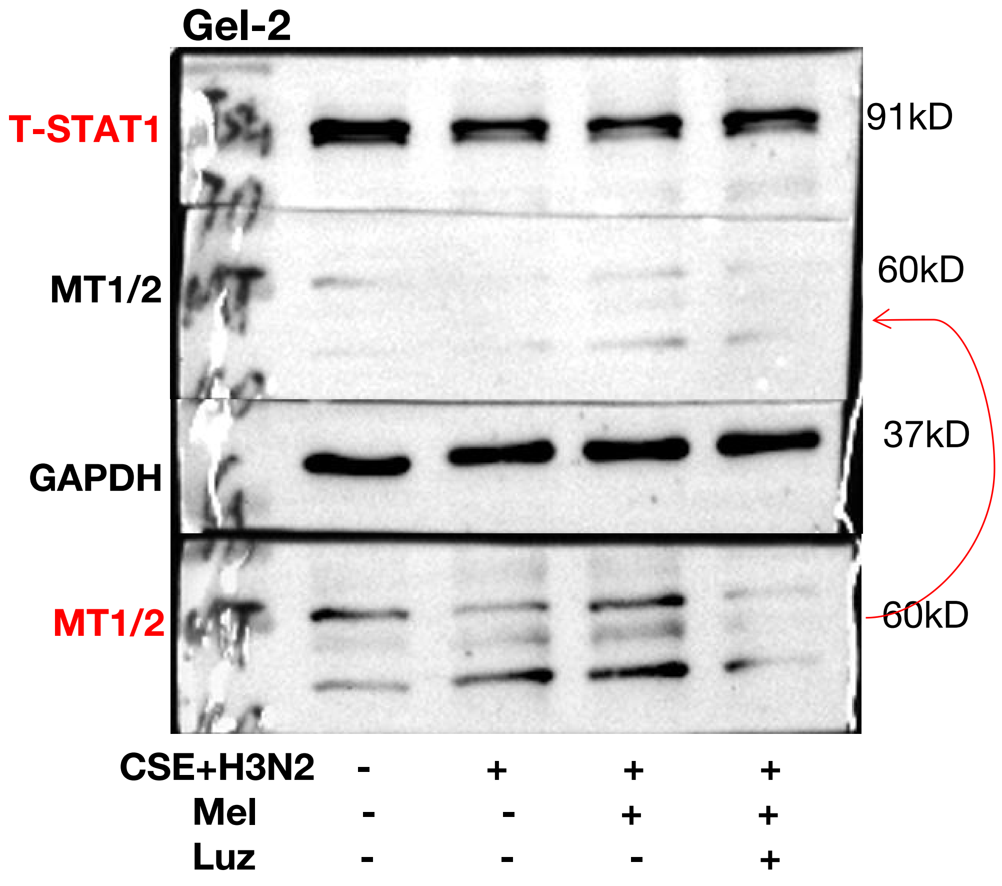

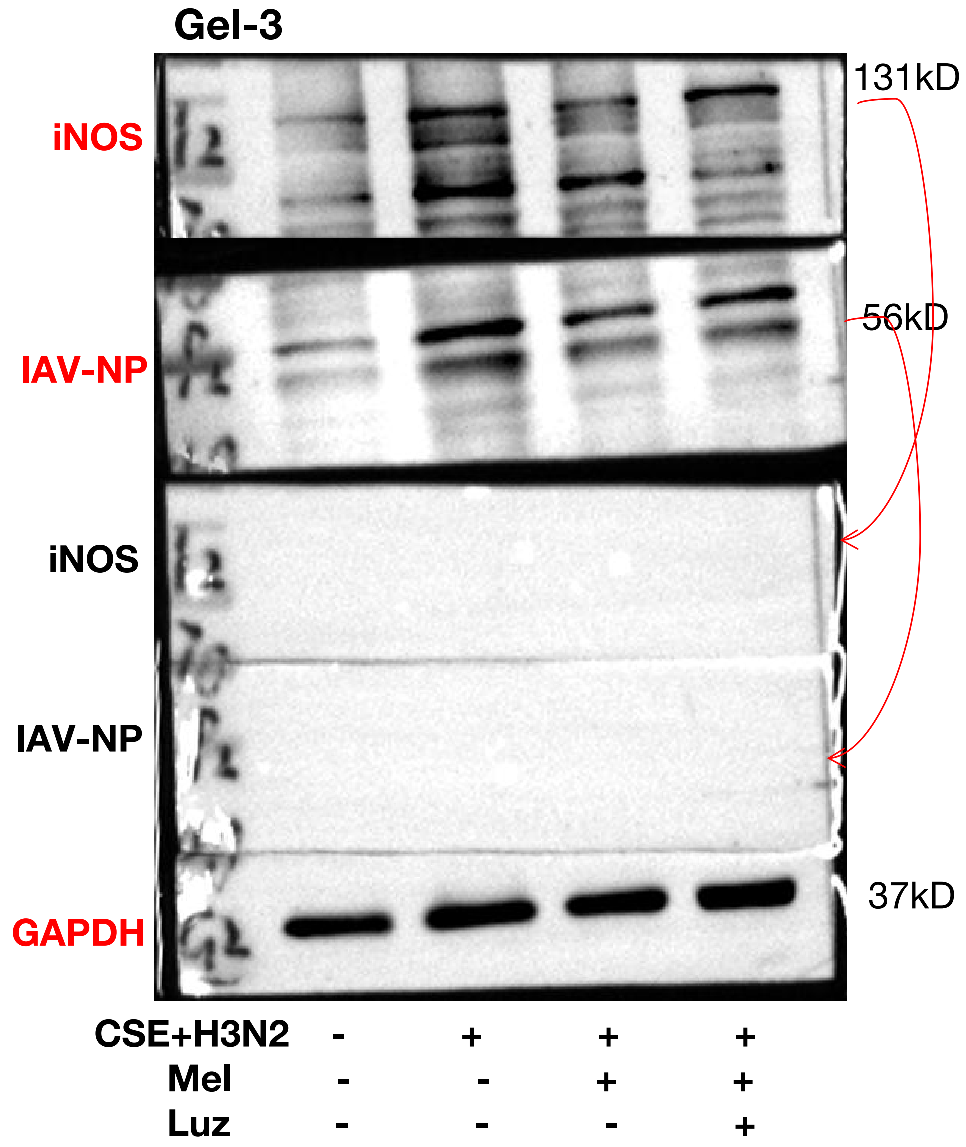

Original full-length gels of Fig 8d + Fig. 9c

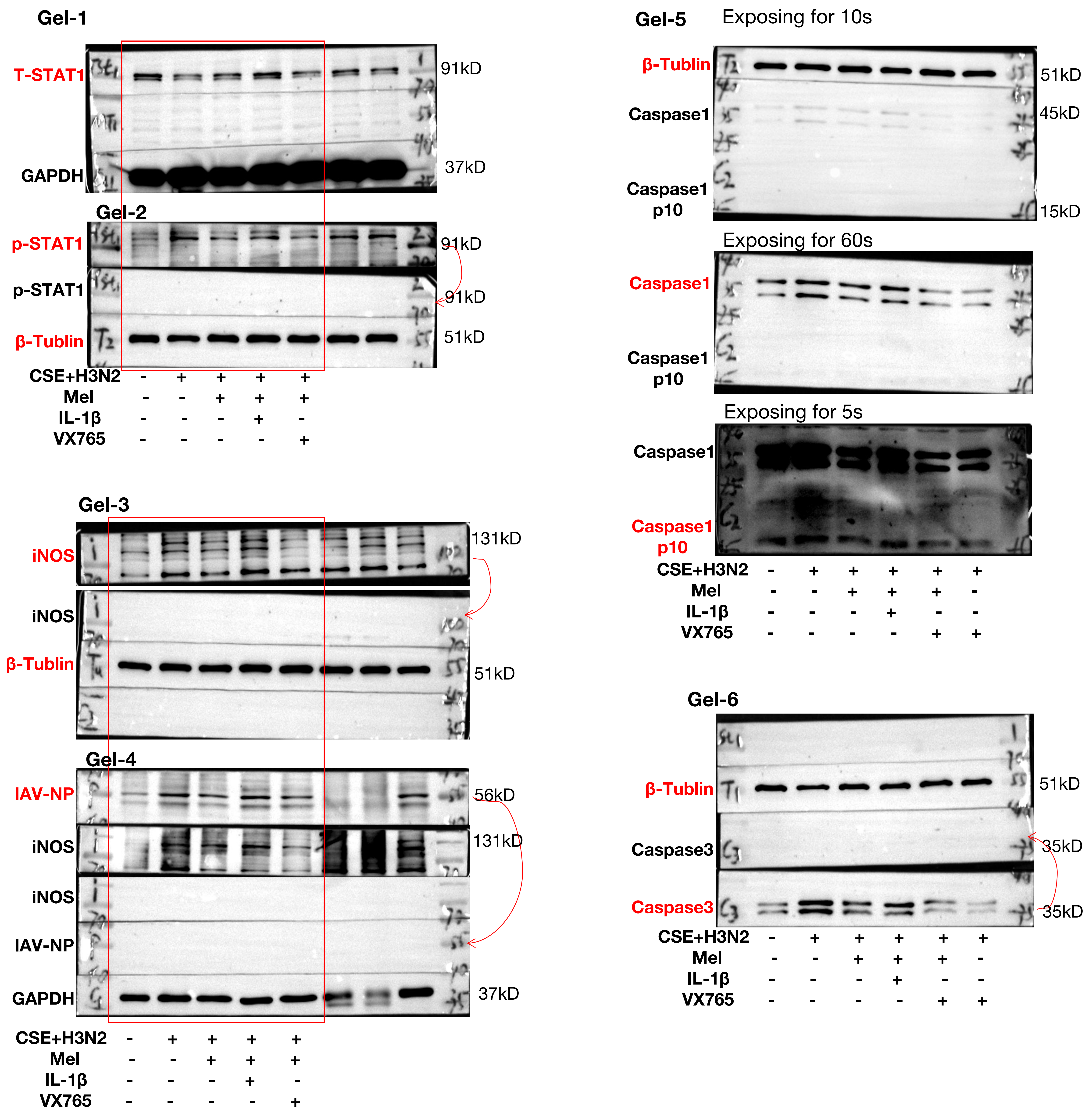

Supplement: Supplementary file 2 — Additional file 2. [file 12931_2024_2815_MOESM2_ESM.pdf]
